# Deleterious effects of formalin-fixation and delays to fixation on RNA and miRNA-Seq profiles

**DOI:** 10.1038/s41598-019-43282-8

**Published:** 2019-05-06

**Authors:** Wendell Jones, Sarah Greytak, Hana Odeh, Ping Guan, Jason Powers, Jasmin Bavarva, Helen M. Moore

**Affiliations:** 1 Q2 Solutions - EA Genomics, Morrisville, NC USA; 2 Kelly Government Solutions, Rockville, MD USA; 3 National Cancer Institute, Bethesda, MD USA; 4 Leidos Biomedical Research, Inc, Frederick, MD USA

**Keywords:** Transcriptomics, Biological techniques

## Abstract

The National Cancer Institute conducted the Biospecimen Pre-analytical Variables (BPV) study to determine the effects of formalin fixation and delay to fixation (DTF) on the analysis of nucleic acids. By performing whole transcriptome sequencing and small RNA profiling on matched snap-frozen and FFPE specimens exposed to different delays to fixation, this study aimed to determine acceptable delays to fixation and proper workflow for accurate and reliable Next-Generation Sequencing (NGS) analysis of FFPE specimens. In comparison to snap-freezing, formalin fixation changed the relative proportions of intronic/exonic/untranslated RNA captured by RNA-seq for most genes. The effects of DTF on NGS analysis were negligible. In 80% of specimens, a subset of RNAs was found to differ between snap-frozen and FFPE specimens in a consistent manner across tissue groups; this subset was unaffected in the remaining 20% of specimens. In contrast, miRNA expression was generally stable across various formalin fixation protocols, but displayed increased variability following a 12 h delay to fixation.

## Introduction

As Next-Generation Sequencing (NGS) technologies have become more affordable, reliable and powerful, they have been increasingly used to determine clinical diagnosis, to guide treatment and predict prognostic outcome (Reviewed in^1^). Although traditionally NGS is conducted using high quality RNA such as that obtained from fresh or frozen specimens, FFPE specimens can also be analyzed by NGS and may be particularly desirable if existing morphological and protein expression data is available along with useful clinical data. Use of FFPE specimens may also allow for the study of archival specimens, as NGS has been shown to be successful in FFPE specimens after as many as 32 years of storage^2^. While RNA is affected by preanalytical handling and FFPE processing, which introduces differences in molecular data obtained with FFPE and frozen specimens, previous studies have shown that expression data from paired FFPE and frozen tissues by NGS can be strongly to very strongly correlated^3–11^. Notably, the expression of 1494 transcripts was found to differ between matched FFPE and frozen specimens^12^. Further, the concordance between FFPE and frozen specimens was affected by RNA quality^3,12^, which is known to be influenced by a number of preanalytical factors including post-mortem interval, delay to fixation (i.e. cold ischemia time), fixation duration, temperature, and storage conditions (Reviewed in^13^). Variation in these preanalytical factors is typical in the clinical laboratories where FFPE biospecimens are generated, but how these factors affect NGS results is unclear.

Although a number of studies have investigated whether nucleic acids extracted from FFPE tissues can be analyzed using NGS platforms, few studies have addressed the effects of formalin-fixation and delayed fixation on the reliability of results of whole transcriptome sequencing and small RNA profiling using case-matched frozen tissues. The National Cancer Institute’s (NCI) Biorepositories and Biospecimen Research Branch (BBRB) developed the Biospecimen Pre-Analytical Variables research program (BPV) to address this and other potential pre-analytical effects of variable FFPE practices. As part of this program, biospecimens collected using strict SOPs were divided shortly after collection and pieces were snap-frozen or formalin-fixed after four different delays to formalin fixation (DTF)^14^. In this paper, we report on differences in whole transcriptome RNA sequencing (RNA-seq) and miRNA expression profiling that are attributable to formalin-fixation and DTF. This study provides guidance on best-practice methodologies for FFPE tissue handling and processing as well as analytical methods to enable the accurate and reliable detection of clinically relevant expression-related endpoints using NGS- based platforms.

## Results of RNA-Seq Analysis

Libraries obtained using Illumina’s TruSeq Total Gold RNA kit represented snap-frozen and FFPE specimens from all 30 tumors and included specimens subjected to a delay to fixation of 1, 2, 3, or 12 h (Supplemental Table 1). The vast majority of libraries were of sufficient quality to conduct 50b PE sequencing and analysis on Illumina HiSeq 2500 instrumentation with a read depth ranging from 50 M to 120 M clusters (Supplementary Table 2), and most specimens sequenced to a depth of ~70 M clusters. The complete dataset is available through dbGaP (phs001639). The RNA-Seq dataset was of very high quality with few outliers and a small set of specimens with noticeable DNA contamination. Only one of the contaminated specimens was deemed an outlier and excluded (specimen #4-4). One lower quality RNA-Seq library (specimen #51-4, colon 1 h DTF) also yielded a poor quality miRNA-Seq library. The main RNA-Seq study thus utilized 148 of the intended 150 specimens for detailed analysis. An overall summary of the gene expression profile via PCA representing all 150 specimens is provided in Supplementary Fig. 1.

**Figure 1:**
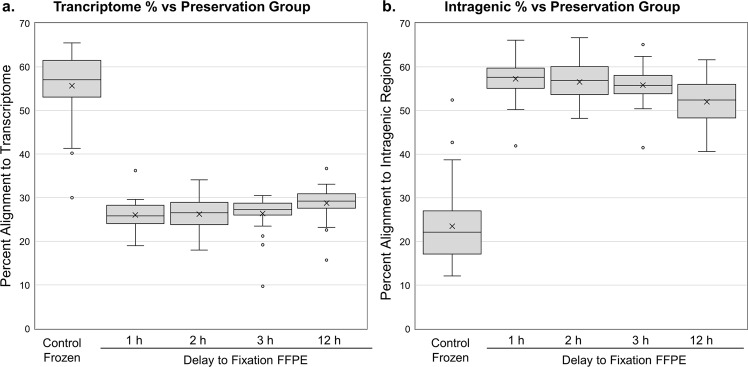
Box plots showing the percentage of RNA-Seq reads that aligned to the transcriptome (**a**) vs. other intragenic alignment including exon-intron chimeras (**b**) in snap-frozen specimens (n = 30) and FFPE specimens fixed after 1 (n = 29), 2 (n = 30), 3 (N = 30) or 12 h (n = 29). The ratio of the percentage of sequences that mapped to the transcriptome vs. other intragenic sequence in FFPE specimens was the inverse of what was observed with snap-frozen specimens. The upper and lower extremes of the box correspond to the first (Q1) and third (Q3) quartiles and the whiskers show the range of the data up to 1.5 times the interquartile range (Q3-Q1). Data more extreme than the range of the whiskers are graphed as individual points. Specimens 4-4 (kidney 12 h DTF) and 51-4 (Colon 1 h DTF).

When comparing results from FFPE and matched frozen tissues, a consistent and dramatic shift in the proportion of reads corresponding to intronic/exonic/untranslated regions in FFPE specimens was detected. FFPE specimens had consistently higher amounts of intronic normalized fragments per thousand bases (NFPK) than matched snap-frozen samples. For a snap-frozen sample processed under the Total RNA protocol, approximately 50–60% of reads mapped to the transcriptome, and another 15–25% aligned to other intragenic regions including exon-intron chimeras (Fig. 1a). However, all formalin-fixed samples displayed an inverse pattern, with only 15–35% of reads aligned to the transcriptome and 50–65% aligned to other intragenic regions (Fig. 1b). Importantly, this finding is not isolated to a few genes, but instead occurred in the majority of genes. These findings may account for the systematic drops in transcriptome alignment^15^ but not genome alignment when using FFPE material.

**Figure 2:**
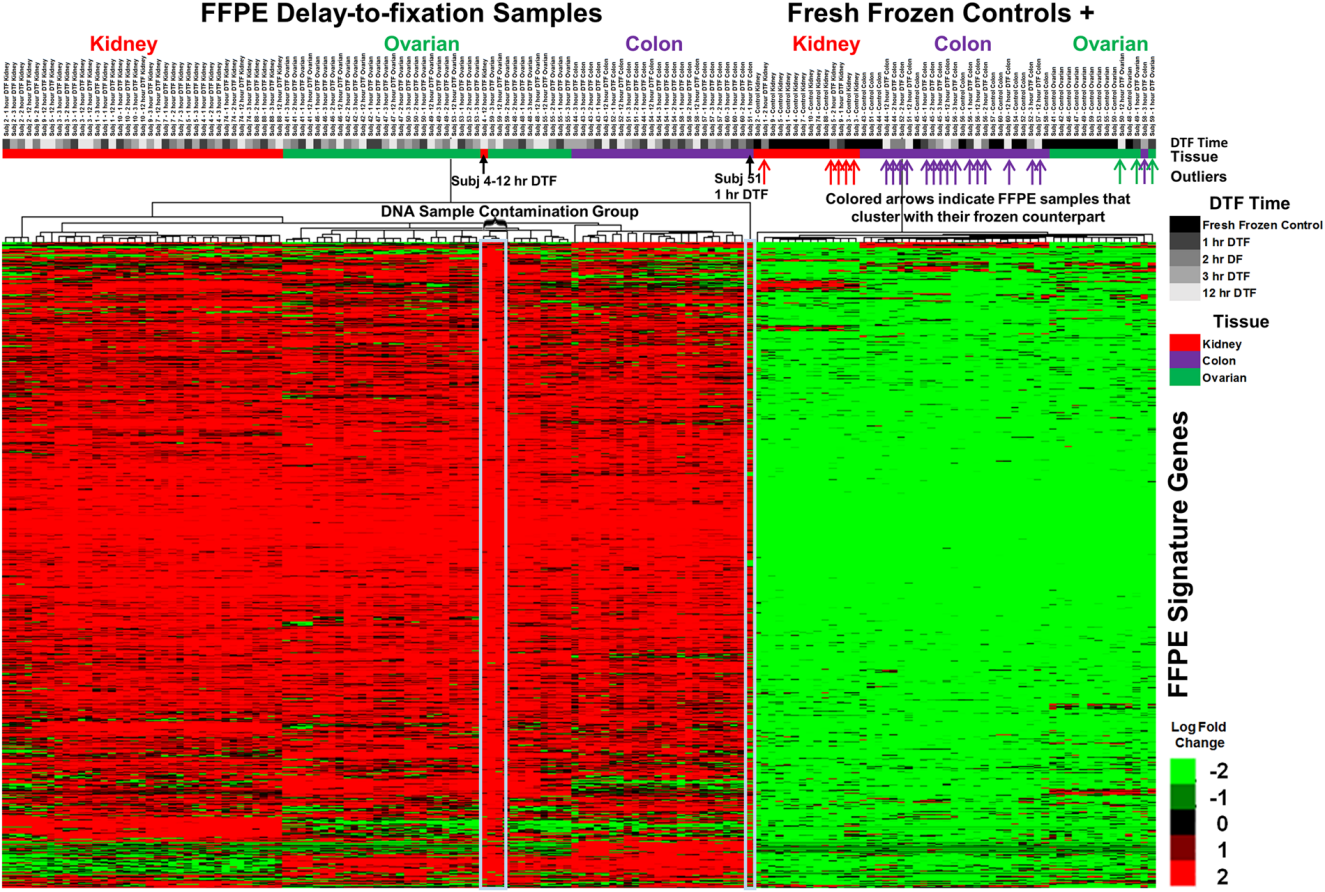
Heat map of median centered log2(RSEM +1) of nearly 1800 genes from FFPE specimens subjected to a 1, 2, 3, or 12 h delay-to-fixation (n = 30 for each timepoint) and case-matched snap-frozen controls (n-30). FFPE specimens displayed higher gene expression than case-matched snap-frozen controls for the majority of FFPE samples examined, regardless of delay-to-fixation. The color bar indicates the tissue/tumor of origin (red-Kidney/renal carcinoma, green-Ovarian/fallopian tube and peritoneal carcinoma, purple-Colon/colon adenocarcinoma). Interestingly, 24 FFPE samples show profiles similar to their snap-frozen control counterparts (indicated by the colored up arrows). Differences between the profiles of these 24 FFPE samples and the remaining FFPE samples were not associated with contributing medical institution, time-to-fixation, density of tumor cells, or even case. Specimens displaying DNA contamination (4-4, 59-4, 59-3, and 59-2) grouped together. Specimens 4-4 (highest level of DNA contamination) and 51-4 (lowest amount of usable material) are indicated by black arrows. The scale indicates log2 log2(RSEM +1) values.

RNA from a large subset of the DTF samples (82%) exhibited differential expression in reference to the matched snap-frozen control, regardless of the duration of the DTF (Fig. 2). Several hundred of these genes were consistently up-regulated in FFPE samples for each DTF time point in comparison to snap-frozen controls. Importantly, the up-regulated gene signature was observed in specimens from all tissue types examined, but it appeared to occur less frequently in colon specimens (24 of the 40 specimens) than other tissues (72 of the 80 kidney and ovary specimens). The magnitude of this increase did not appear to be affected by the DTF time point, but instead appeared to be attributable to fixation alone. Interestingly, when these genes were subjected to enrichment analysis, the pathways typically associated with stress or hypoxia were absent (Supplemental Table 3). Instead, DNA/chromatin packaging and nucleosome organization pathways were enriched.

## RNA-Seq Differential Gene Lists

We compared lists of genes identified as differentially expressed between tumor/tissue types in snap-frozen specimens with lists generated for 1 or 12 h DTF FFPE specimens. Differential expression was defined as having a greater than 1.5-fold difference in expression between tissue/tumor types and a t-test significance of P < 0.001 (based on the Microarray Quality Control (MAQC) study^16^ finding that use of fold-change in addition to P-values increased the reproducibility of genes lists). As shown in Table 1, overlap of genes differentially expressed between tumor types among snap-frozen controls and the 1 hour and 12 hour DTF specimens exceeded 70% in all cases, regardless of the pipeline used for the differential analysis. As some variability is assumed due to variation in expression measurement, instability of variance estimates, and other factors, this indicates that DTF and preservation method had little or no effect on detection of gene expression. In addition, the magnitude of variation in expression of individual genes, as well as the rank-ordering of genes by expression variation, were strongly correlated between DTF and snap-frozen controls for all tissues examined, with Spearman rank correlation coefficients ranging between ρ = 0.84 and ρ = 0.88.

**Table 1:** Size and overlap of differential list comparisons between renal carcinoma (Kidney, n = 10), colon adenocarcinoma (Colon, n = 10) and fallopian tube and peritoneal carcinoma (Ovary, n = 10) specimens.

	Tumor Tissue Comparison	Preservation Groups Analyzed: Group 1 and Group 2	Sample sizes for tissues compared Group 1(n_1_ and n_2_)	# of genes differentially expressed between tissues Group 1	Sample sizes for tissues compared Group 2 (n_1_ and n_2_)	# of genes differentially expressed between tissues Group 2	% overlap in the differential gene lists
RSEM and t-test	Kidney vs. Colon	Frozen and 1 hr DTF	10 vs. 10	3981	10 vs. 9	4312	84%
	Kidney vs. Colon	Frozen and 12 hr DTF	10 vs. 10	3981	9 vs. 10	3675	75%
	Kidney vs. Colon	1 hr and 12 hr DTF	10 vs. 9	4312	9 vs. 10	3675	82%
	Ovarian vs. Kidney	Frozen and 1 hr DTF	10 vs. 10	2160	10 vs. 10	2133	75%
	Ovarian vs. Kidney	Frozen and 12 hr DTF	10 vs. 10	2160	10 vs. 9	2213	77%
	Ovarian vs. Kidney	1 hr and 12 hr DTF	10 vs. 10	2133	10 vs. 9	2213	80%
	Ovarian vs. Colon	Frozen and 1 hr DTF	10 vs. 10	3303	10 vs 9	3506	83%
	Ovarian vs. Colon	Frozen and 12 hr DTF	10 vs. 10	3303	10 vs 10	2875	74%
	Ovarian vs. Colon	1 hr and 12 hr DTF	10 vs. 9	3506	10 vs. 10	2875	83%
Feature counts and DESeq2	Kidney vs. Colon	Frozen and 1 hr DTF	10 vs. 10	9385	10 vs. 9	9585	77%
	Kidney vs. Colon	Frozen and 12 hr DTF	10 vs. 10	9385	9 vs. 10	8855	76%
	Kidney vs. Colon	1 hr and 12 hr DTF	10 vs. 9	9585	9 vs. 10	8855	82%
	Ovarian vs. Kidney	Frozen and 1 hr DTF	10 vs. 10	8493	10 vs. 10	8738	77%
	Ovarian vs. Kidney	Frozen and 12 hr DTF	10 vs. 10	8493	10 vs. 9	7723	75%
	Ovarian vs. Kidney	1 hr and 12 hr DTF	10 vs. 10	8738	10 vs. 9	7723	82%
	Ovarian vs. Colon	Frozen and 1 hr DTF	10 vs. 10	6746	10 vs 9	6654	75%
	Ovarian vs. Colon	Frozen and 12 hr DTF	10 vs. 10	6746	10 vs 10	7018	73%
	Ovarian vs. Colon	1 hr and 12 hr DTF	10 vs. 9	6654	10 vs. 10	7018	78%

All DTF specimens are FFPE.

## Linear Models of RNA-Seq Profiles

To examine and quantify the effects of tissue type, preservation protocol, contributing medical institution, and patient as well as their primary interactions, we constructed linear models for individual genes. This linear model was more powerful than the group comparison test as it used all 148 specimens per gene whereas each group comparison test only used up to 20 specimens (assuming no outliers were removed). To facilitate interpretation of linear models, we restricted the set of genes modeled to the 18,217 genes that had normalized mean expression levels of at least 16 (roughly corresponding to a mean raw count of five depending on the original sample read depth) and determined the effect size by calculating the relative least squares estimate of each factor level. We created gene lists that identified differences in expression with an effect size greater than 1.5-fold and unadjusted p-values less than 0.001 between preservation groups using linear contrasts.

Very few genes had significant interactions between factors in a first pass analysis (<0.1%) and, therefore, factor interaction terms were omitted. As summarized in Table 2 and shown in Supplemental Fig. 2, tissue type affected the measured expression of the largest number of genes (6210–7570 genes, 34.10–41.6% of tested genes). Comparatively, fewer genes had differences in measured expression between snap-frozen and FFPE preserved specimens, regardless of DTF time point (3138–3758 genes, 17.20–20.60% of tested genes) or contributing medical institution (463–1023 genes, 2.50–5.60% of tested genes). Duration of the delay-to-fixation in FFPE samples impacted only 22–45 genes (0.10–0.20% of tested genes) making it the least influential of the factors tested. The fact that the specific DTF time was one of the least influential linear model factors explaining differential expression variation was one of the more surprising study results.

**Table 2:** The number of differentially expressed genes relative to primary contrasts in the RNA study.

Contrasts Tested	Freq. of RNA Diff Exp	% of genes tested	Freq. of RNA Diff Exp	% of RNA tested
Presv. Group Contrast - Ctl vs. 1 hr DTF	3758	20.60%	16	4.80%
Presv. Group Contrast - Ctl vs. 2 hr DTF	3725	20.40%	21	6.30%
Presv. Group Contrast - Ctl vs. 3 hr DTF	3705	20.30%	17	5.10%
Presv. Group Contrast - Ctl vs. 12 hr DTF	3138	17.20%	19	5.70%
Presv. Group Contrast - 1 hr vs. 12 hr DTF	22	0.10%	2	0.60%
Presv. Group Contrast - 2 hr vs. 12 hr DTF	22	0.10%	3	0.90%
Presv. Group Contrast - 3 hr vs. 12 hr DTF	45	0.20%	2	0.60%
Med Inst Contrast - BMC-EU	1023	5.60%	30	9.00%
Med Inst Contrast - BMC-UNM	497	2.70%	15	4.50%
Med Inst Contrast - BMC-UPMC	676	3.70%	28	8.40%
Med Inst Contrast - EU-UNM	954	5.20%	35	10.40%
Med Inst Contrast - EU-UPMC	634	3.50%	16	4.80%
Med Inst Contrast - UNM-UPMC	463	2.50%	30	9.00%
Tissue Contrast - Colon-Kidney	7570	41.60%	191	57.00%
Tissue Contrast - Colon-Ovary	6210	34.10%	136	40.60%
Tissue Contrast - Kidney-Ovary	7397	40.60%	199	59.40%

Lists were determined by contrasts using one overall linear model, n = 148.

The linear model also demonstrated another interesting facet. Genes that were found to be differentially expressed in the simple group comparison between tissues under one preservation condition but not another (Table 1) were also found to be differentially expressed in the same manner using the linear model. Therefore, the genes specific to each simple group comparison performed previously were not false positives, but rather true positives within that preservation group and false negatives in the other preservation group. Thus, this implies there is an increase in statistical power associated with examining the data as a whole which reduces false negatives that occur when simple direct comparisons of only two groups are considered. Further, individual snap-frozen control- and FFPE-based tissue comparisons provided high positive predictive value (PPV) gene lists for differential expression.

**Figure 3:**
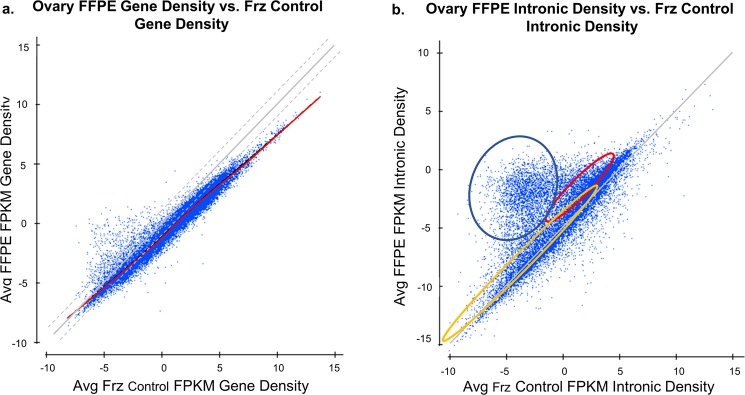
(**a**) General monotonicity in the log FPM of the FFPE results in Ovarian specimens (n = 40) vs. the Log FPKM of the snap-frozen controls (n = 10) at the gene level for the exonic and UTR regions (ie, the transcriptome). There is a noticeable compression in the slope of about 15%. Pearson r = 0.97. (**b**) Clusters of density of fragments (log FPKM) by gene in the intronic regions of FFPE samples (Y-axis) vs. the density of fragments (log FPKM) in the intronic regions of snap-frozen controls (x-axis). Each colored ellipse (Blue, Red, Orange) contains approximately 25–30% of the genes graphed. Pearson r = 0.74.

When we investigated the high concordance in differential expression further, we observed a strong positive correlative relationship (Pearson r = 0.97) between the relative density of material in coding and UTR regions (using measures such as FPKM) of snap-frozen and FFPE material (Fig. 3a), but the relationship in intronic regions was less correlative (Pearson r = 0.74) and more complex (Fig. 3b). This data may have strong ramifications for estimating splice variation in FFPE specimens from short reads as the intron of one isoform may be an exon for another. Figure 3b suggests that the large amounts of extraneous-spliced material in FFPE specimens will prove difficult for quantification methods at the transcript level for multiply-spliced genes, affecting roughly two-thirds of the genes that are multiply spliced.

## Results of the miRNA-Seq Analysis

**Figure 4:**
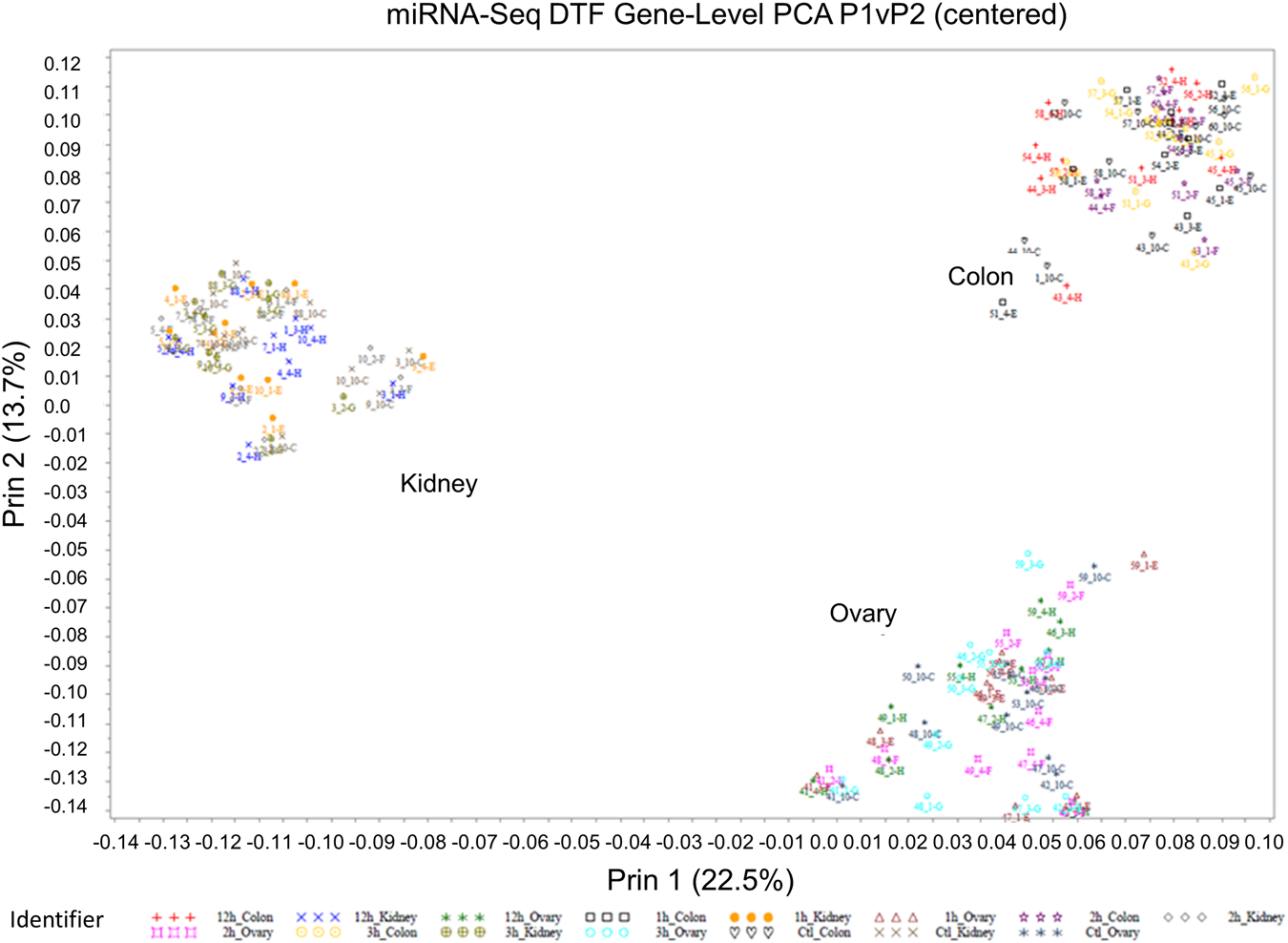
First two principal components of miRNA expression analysis in the BPV Study. Samples are colored by tissue and by the preservation group: FFPE with 1 (n = 29), 2 (n = 30), 3 (n = 30), or 12 h (n = 30) DTF or matched snap-frozen controls (n = 30). Renal carcinoma (kidney), colon adenocarcinoma (colon) and fallopian tube and peritoneal carcinoma (ovary) specimens clustered separately in this plot, but there was no clustering by DTF. One colon specimen with a 1 h DTF (Sample 51-4) was omitted.

All 150 samples yielded RNA of sufficient quality and thus were utilized for miRNA analysis (Supplemental Table 4). DTF conditions had a surprisingly mild impact on the mean miRNA expression measurements overall. The miRNA expression profiles using the top two principal components clearly segregated by tissue type and not preservation protocol (Fig. 4). In fact, there was no clear bias due to preservation protocol in the top five principal components.

DTF had a more general impact on the quality of miRNA data. miRNA detection levels were consistently negatively correlated with DTF time across all three tissue types (Supplemental Fig. 3a). Likewise, the 75^th^ percentile count (upper quartile) was negatively correlated with DTF time with the typical upper quartile value decreasing by approximately 75 counts (or 15%) for 12 h DTF samples when compared to snap-frozen controls. This decline in counts is most likely due to the increased presence of degradation products of other RNAs that are within the selected RNA nucleotide size range (<34 bases). Quality review of the adapter-clipped library sequences showed a notable increase in the proportion of library material that was greater than 25 bases in length for 12 hour DTF samples when compared with other DTF time points.

Unlike RNA, DTF had a larger impact on the variation of miRNA-measured expression within a tissue for a specific preservation group. In general, specimens with a 12 hour DTF displayed more variation by miRNA across samples when compared to snap-frozen controls (10% to 30% higher across tissue types, p < 10E-06 two sided, see Supplemental Fig. 3b), but the magnitude of increase in variability was miRNA species-dependent. Consequently, the correlation of rank ordering for variation in miRNA species expression among DTF specimens and snap-frozen controls was significantly lower than observed with the RNA-seq data (Spearman rank correlation coefficients for miRNA variation for each DTF time point and snap-frozen control when separate by tissue, ρ = 0.52–0.76). The change in miRNA variation rank order between snap-frozen control and FFPE preservation methods was particularly pronounced for Colon and Ovary 12 h DTF specimens (Supplemental Fig. 4). The majority of variation changes were increases in variation, which in turn led to reduced power to detect differentially expressed miRNA in specimens subjected to a DTF, particularly in the case of a 12 h DTF.

When lists of miRNA differentially expressed between tissue types were compared across preservation methods, there was >70% overlap in differential miRNA lists generated for snap-frozen control samples versus the two DTF extremes (1 h and 12 h delays), with only one exception. However, there was a tendency to detect fewer differentially expressed miRNA in specimens with a 12 h DTF. This coincided with the lower detection level associated with 12 h DTF. Regardless, similar final results can be expected from differential analysis experiments using tissues subjected to a delay-to-fixation of up to a 12 h.

## Discussion

We found that differential analysis of miRNA and RNA from FFPE specimens exposed to various delays-to-fixation by NGS can yield reliable and reproducible results so long as comparative analysis is carefully and consistently performed and specimens are limited to those derived from similar protocols (preservation and processing). In particular, when directly comparing miRNA or mRNA expression in formalin-fixed samples vs. snap-frozen samples, differences due to preservation method alone can be anticipated. These differences are due to a large set of RNA (in the thousands) and a smaller number of miRNA whose expression measurements are altered in a generally consistent manner by formalin-fixation across tissue types. As reported previously, formalin-fixation also led to a large increase in off-target reads associated with intronic regions^5,10,12,17^ even in specimens with comparable exome expression data to a snap-frozen counterpart. Thus, gene expression is more difficult to quantify without enrichment for exonic material using FFPE tissue in comparison to snap-frozen specimens at the gene level and especially the isoform level. Consistent with qRT-PCR results in these specimens^18^ DTF had little effect on measured RNA expression, but there was an increase in the abundance of small RNA fragments (<34 bases), likely due to RNA degradation, and a noticeable increase in the variability of miRNA expression measurements for a subset of miRNAs.

Although systemic differences in levels of several thousand transcripts were observed between most formalin-fixed specimens and snap-frozen matched specimens, such effects were not observed in 20% (24 of 120) of DTF specimens (Fig. 2). Specimens from colon were more likely not to display the formalin-fixation associated transcriptome changes (16 of 40) than specimens from other tissues (8 of 80), but there was no clear interaction between these changes and DTF time-point or contributing medical institution. As formalin-fixation had a less discernible impact on relative miRNA levels (mostly affecting variability), it’s not possible to discern if the effects of formalin-fixation on miRNA were different in specimens in which formalin-fixation associated transcriptome changes were absent than in those in which they occurred. It is interesting to note that these specimens were putatively handled in a very consistent manner using well-defined standard operating procedures^14^; thus, any observable difference cannot be attributed to variations in specimen processing nor can they be attributed to between-patient variability as effects were observed for some but not all specimens from the same tumor. Importantly, the FFPE specimens that lacked the induced formalin-associated upregulated expression also had the same higher percentage of intronic reads observed in all FFPE specimens (relative to snap-frozen controls), indicating this phenomenon is not due to a labeling error. Analysis of the genes upregulated in the majority of FFPE specimens revealed enrichment of genes with roles in nucleosome and chromatin assembly, as well as histone core genes.

The cause behind the higher percentage of intronic reads in FFPE specimens versus snap-frozen specimens is unclear. Previously, this has been attributed to differences in library construction methods^5,10^, but in this study identical methods were used. We similarly discounted potential differences in the likelihood of sequencing nucleic acid material from the nucleus relative to the cytoplasm, as this would be expected to increase DNA contamination and levels of snoRNA or XIST expression (whose molecules typically reside only in the nucleus) in FFPE samples relative to their matched snap-frozen counterparts and this was not observed. The increase in intronic reads could also be due to formalin interference with proteins that perform splicing, allowing transcription of a large amount of immature RNA to go unspliced, but formalin-fixed specimens had comparable splice junction rates (relative to the amount of exonic material) to that of their matched snap-frozen counterparts. A remaining conjecture is that the formalin fixation process increases levels of proteins bound to intronic regions and thus prevents the intronic regions of these genes from being digested quickly.

We previously reported the results of nucleic acid quality assays on the biospecimens from the NCI BPV study, finding limited effects of DTF on RNA quality metrics^14^. Consistent with these results, DTF had little to no effect on RNA-Seq results. However, DTF of 12 h increased the variability in miRNA-Seq data and as previously reported resulted in decreased DV200 in the kidney specimens^14^. This lack of effect on RNA-Seq results is interesting as it may allow for more flexibility in terms of time between surgical excision and fixation. However, the shortest DTF explored in this study was 1 h; therefore, we cannot rule out the possibility that particularly labile mRNA may be affected by delays shorter than 1 h. Previous studies using microarrays have reported significant differences in gene expression when snap-freezing was delayed by 15 min rather than 0 min^19^, 20 min versus 5 min^20^, 30 min rather than 5 min^21^ or 45 min versus 10 min^22^, and when RNAlater preservation was delayed by 30 min^23^, but other microarray studies found no significant effects of 40 min^24^ and 5 min versus 2 h^25^ cold ischemia. Further, an increase in the number of short RNA fragments was noted after a DTF of 12 h and is consistent with the decreased DV200 in these kidney specimens after a delay of 12 h^14^. This is presumably due to degradation of RNAs and consistent with findings of reduced RIN with ischemia of 45 min^26^ or 90 min versus 10 min^27^. The increase in short RNA fragments results in a species-dependent decrease in miRNA counts and increase in miRNA count variability. Consequently, while RNA-seq may be possible in specimens subjected to a 12 h DTF, use of these specimens for miRNA-seq would result in less power in the detection of small changes in expression despite only limited observed effects on RNA quality metrics^14^ unless sequencing depth was increased to compensate.

We conclude that FFPE specimens preserved using a consistent protocol are likely suitable for gene expression and miRNA profiling. However, it may be necessary to adopt library preparation protocols that enrich for exonic material and ensure that delays to fixation are limited to 3 hours or less. Primary factors affecting the analysis of RNA isolated from FFPE specimens include a very consistent increase in off-target library material and increases in expression of specific genes. One way to compensate for the increase in off-target reads is enriching for target exonic material during library preparation or by increasing sequencing depth 2-fold relative to snap-frozen specimens. Similarly, while less impacted by FFPE than whole transcriptome sequencing, miRNA sequencing may require a 10–20% increase in standard read depth for FFPE specimens subjected to a delay to fixation of 3–12 h to compensate for the increase in off-target material observed with longer DTF. It is important to note, that despite adherence to strict protocols and detailed specimen annotation, the effects of formalin-fixation on the measured expression of select genes and isoforms (3–4 k in number) in 80% of specimens remains problematic, rendering their absolute and even relative quantification unreliable until more is known about the associated mechanism.

## Methods

### Specimens

The effect of DTF on RNA and miRNA-Seq results were investigated using specimens from 30 tumors including 10 renal clear cell carcinomas (Kidney), 10 serous ovarian carcinomas (Ovary), and 10 colon adenocarcinomas (Colon) collected at 4 medical centers using Institutional Review Board (IRB)-approved protocol was used by each medical site for the collection of human biospecimens for research purposes in accordance with the Helsinki Declaration of 1975, as revised in 1983 [Emory University IRB00045796 (approved 3/21/2013); University of New Mexico IRB00000591 (approved 06/28/2012); University of Pittsburgh IRB0106147 (approved 05/28/2014), IRB0411047 (approved 07/18/2014), IRB09502110, IRB0506140 (approved 5/28/2014), and IRB056140 (approved 06/19/2014); and Boston Medical Center IRB00000376 (approved 02/05/2014)]. Informed written consent was obtained from each patient. Further details regarding consent, acquisition and processing are detailed in a previous publication^14^. Each tumor was divided into six samples one of which was snap-frozen in a prechilled cryosette in liquid nitrogen vapor soon (<1 hr) after resection and stored in liquid nitrogen. The remaining samples were subjected to 1, 2, 3 or 12 h of cold ischemia in a humidified chamber at room temperature prior to fixation in 10% neutral buffered formalin (NBF) for 12 hours and paraffin-embedding. FFPE blocks were stored for 3 to 18 months prior to extraction.

### RNA and miRNA isolation

All nucleic acid extractions were performed by the Van Andel Research Institute (VARI). RNA was extracted from snap-frozen and FFPE specimens using QIAsymphony kits. Briefly three freshly cut 10 µm thick FFPE sections were placed in deparaffinization solution at 56 °C, then digested with proteinase K at 56 °C for 15 min before loading into the QIAsymphony sample drawer for RNA extraction. Similarly, snap-frozen specimens were disrupted using the TissueLyser II homogenizer and supernatants were loaded into the QIAsymphony sample drawer. Specimen quality was assessed as part of a previous report^14^ and for relevant specimens is available in Supplemental Table 1.

### RNA-Seq - methods

RNA samples were converted into cDNA libraries using the Illumina TruSeq Stranded Total RNA sample preparation kit (Illumina \# RS-122-2303) and sequenced using a HiSeq 2500. Briefly, Total RNA samples were concentration normalized, and ribosomal RNA (rRNA) was removed using biotinylated probes that selectively bind rRNA species. This process preserved mRNA and other non-coding RNA species including long intergenic noncoding RNA (lincRNA), small nuclear RNA (snRNA) and small nucleolar RNA (snoRNAs). The resulting rRNA-depleted RNA was fragmented using heat in the presence of divalent cations. Fragmented RNA was converted into double-stranded cDNA, with dUTP utilized in place of dTTP in the second strand master mix. A single ‘A’ base was added to the cDNA along with forked adaptors that included index, or barcode, sequences that were attached via ligation. The resulting molecules were amplified via polymerase chain reaction (PCR). During PCR the polymerase stalled when a dUTP base was encountered in the template. Since only the second strand included the dUTP base, the first strand was the only viable template, thereby preserving the strand information. Final libraries were quantified, normalized and pooled. Pooled libraries were bound to the surface of a flow cell and each bound template molecule was clonally amplified up to 1000-fold to create individual clusters. Four fluorescently labeled nucleotides were then flowed over the surface of the flow cell and incorporated into each nucleic acid chain. Each nucleotide label acts as a terminator for polymerization, thereby ensuring that a single base was added to each nascent chain during each cycle. Fluorescence was measured for each cluster during each cycle to identify the base that was added to each cluster. The dye was then enzymatically removed to allow incorporation of the next nucleotide during the next cycle.

#### Processing pipeline for RNA-Seq data

Multiplexed FASTQs were created using Illumina’s CASAVA 1.8.2 software. Reads were demultiplexed using fastq-multx and quality-trimmed using fastq-mcf [ver. EA-Utils 1.04.773], which removed low quality bases from the ends of reads, low quality reads, and residual Illumina adapters. We used EA Genomics v9 RNA pipeline for RNA-Seq quantitation. Briefly, the human transcriptome is defined by University of California Santa Cruz (UCSC), but with the addition of the Ensembl lincRNAs. We implemented STAR v2.4 as an aligner specifically designed for RNA-Seq. Post-alignment, the resulting bam file was fed into the RNA-Seq quantification software RSEM, version 1.2.14. For this study, we only utilized gene-level results that were log(count +1) based with normalization via upper quartile methods of generally detected genes. Hg19 was used as the reference genome.

#### Post-quantification analysis

Post-quantification, we further characterized reads into those which aligned to UTRs, and those which aligned to intragenic regions. In both cases we used the SAMPLE.star.bam, coupled with custom region definitions. Using these definition files, we parsed the alignments found in the SAMPLE.star.bam, and counted the number of alignments to each region of interest. We counted reads per UTR per gene. In a similar manner we characterized intragenic reads as those that fall between the 5′ and 3′ end of a gene, but not within known exons or the UTRs. For this analysis, we defined the intragenic regions separately and performed a special alignment and quantification using RSEM to determine intragenic counts per gene.

### miRNA-Seq - Methods

Protocol for laboratory processing: Total RNA samples were converted into indexed cDNA sequencing libraries using Illumina’s TruSeq Small RNA sample preparation kit (Illumina \# RS-200-0012). Starting with ~200 ng of total RNA, a single stranded adenylated DNA adapter was added to the 3′ hydroxyl group using T4 RNA Ligase 2 deletion mutant. The T4 RNA ligase 2 deletion mutant prevents ligation of the adapter to the 5′ end due to the absence of ATP. A 5′ adapter was then added to the 5′ phosphate using T4 RNA Ligase in the presence of ATP. Following adapter ligation, single stranded cDNA was created by a reverse transcription reaction. The cDNA was then PCR amplified using a common sequencing primer and an indexed primer that is unique to each sample. The cDNA libraries were analyzed for quality and fragment size ranges using the Agilent 2200 Tapestation (D1000 Screentape, Agilent \# 5067–5582). Libraries were then size-selected, retaining fragments of between roughly 125–160 bp, using BluePippin (3% cassettes, Sage Science \# BDF3010), resulting in a mean library size about approximately 135 bp. The final libraries were then quantified by qPCR (KAPA Library Quant Kit, KAPA Biosystems \# KK4824), and normalized to 2 nM in preparation for sequencing on a HiSeq 2500.

Processing pipeline for miRNA-Seq data: Multiplexed FASTQs were created using Illumina’s CASAVA 1.8.2 software. Reads were demultiplexed using fastq-multx and quality-trimmed using fastq-mcf [ver. EA-Utils 1.04.773], which removed low quality bases from the ends of reads, low quality reads, and residual Illumina adapters. Removal of this residual adapter is particularly important for miRNA analysis, as miRNAs are shorter than the overall read length, and therefore the adapter sequence should be present on nearly every sequencing read. Post-clipping, it was ensured that the average read length of the remaining sequence was in line with typical small RNA sizes. After clipping, a basic alignment of miRNAs to those found in the mirBase database, allowing for 1 base mismatch, was performed. The reads associated with each miRNA were counted and normalized using upper quartile methods.

### Statistical methods

In general, principal component analysis used all samples including outliers and was performed using SAS v9.4 PROC PRINCOMP (no further options). The analysis was conducted on log-transformed signal, centered by gene.

Hierarchical clustering in general used correlation as a measure of similarity and centroid-based linkage.

Simple differential analysis of groups using the RNA-Seq data was performed using two methods. In one method, the well-known t-statistic with a mildly stringent unadjusted p-value of 0.001 was combined with a fold change threshold of 1.5 to generate comparator lists. In a comparator method, we used the RNA-Seq differential method DESeq2^28^ with the same fold change threshold and a (multiple testing) corrected or adjusted p-value of 0.01. The linear model for the RNA-Seq analysis utilized 148 of the original 150 samples (specimens 4-4 and 51-4 omitted). The linear model was performed using SAS v9.4 PROC MIXED with subject as a random effect and terms for fixed effects of tissue type, collection site, and preservation protocol. Only effects with unadjusted p < 0.001 were kept for meta-analysis across genes.

We used Levene’s test (two-sided) for homogeneity of variance (SAS) when examining variation in miRNA expression by protocol.

## Supplementary information

Supplementary Data 1

## Data Availability

SOPs generated for the study are available through the publicly available at https://biospecimens.cancer.gov/programs/bpv/bpv_sops.asp as well as through the Biospecimen Research Database SOP repository (hhttps://brd.nci.nih.gov/brd/sop-compendium/show/1062). Study documents as well as the meta-data on variables are available publicly through dbGaP (#phs001304). Data on individual specimen quality metrics is available in a previous publication^14^ and for specimens used in this analysis in Supplemental Table 1. Software used in specimen analysis is commercially or freely available and detailed in the methods section. RNA and miRNA sequencing data and phenotypic data used for Tables 1 and 2 and Figs 1–4 are available under controlled access from dbGaP (#phs001639).
