# Supplementary material for: Deleterious effects of formalin-fixation and delays to fixation on RNA and miRNA-Seq profiles
Source: Sci Rep. 2019 May 6;9:6980. doi: 10.1038/s41598-019-43282-8 (PMC6502812; doi:10.1038/s41598-019-43282-8)
Supplement: Supplementary file 1 — Supplementary Data 1 [file 41598_2019_43282_MOESM1_ESM.pdf]

Deleterious effects of formalin-fixation and delays to fixation on RNA and miRNA-Seq profiles

Wendell Jones[1*], Sarah Greytak[2], Hana Odeh[3], Ping Guan[3], Jason Powers[1], Jasmin Bavarva[4,] Helen M. Moore[3]

[1] Q^2 Solutions - EA Genomics

[2] Kelly Government Solutions

[3] NCI

[4] Leidos

Supplementary Figures

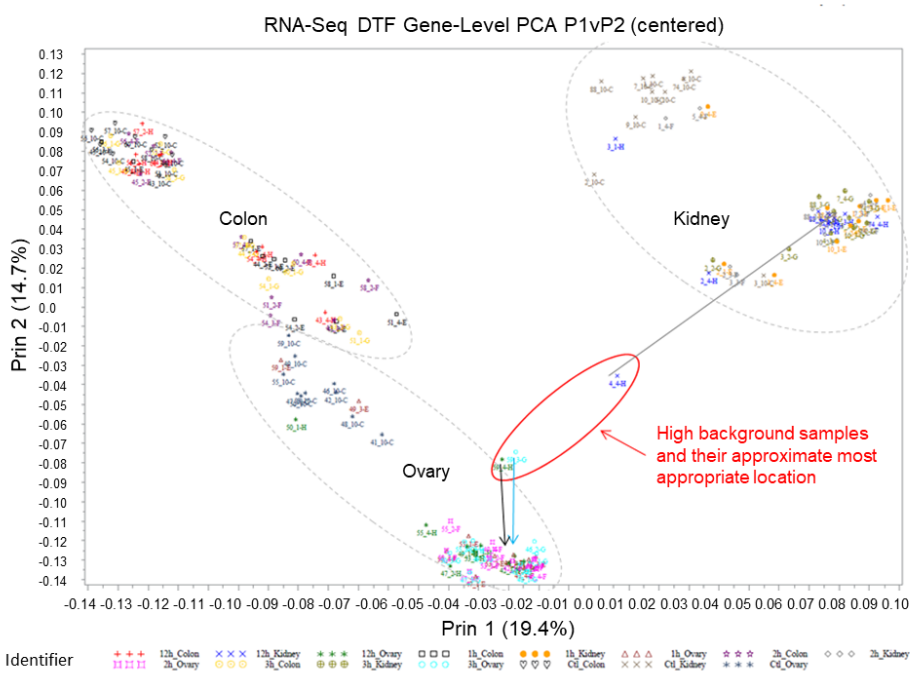

Supplementary Figure 1. First two principal components of RNA-Seq analysis in the BPV Study. Samples are colored by tissue and by the preservation group: FFPE with 1, 2, 3, or 12 h DTF (n=30 each) or matched snap-frozen controls(n=30). Renal carcinoma (kidney), colon adenocarcinoma (colon) and fallopian tube and peritoneal carcinoma (ovary) specimens clustered by tissue of origin. Three samples with higher backgrounds are highlighted; one of these (4-4H) was omitted from any further analysis.

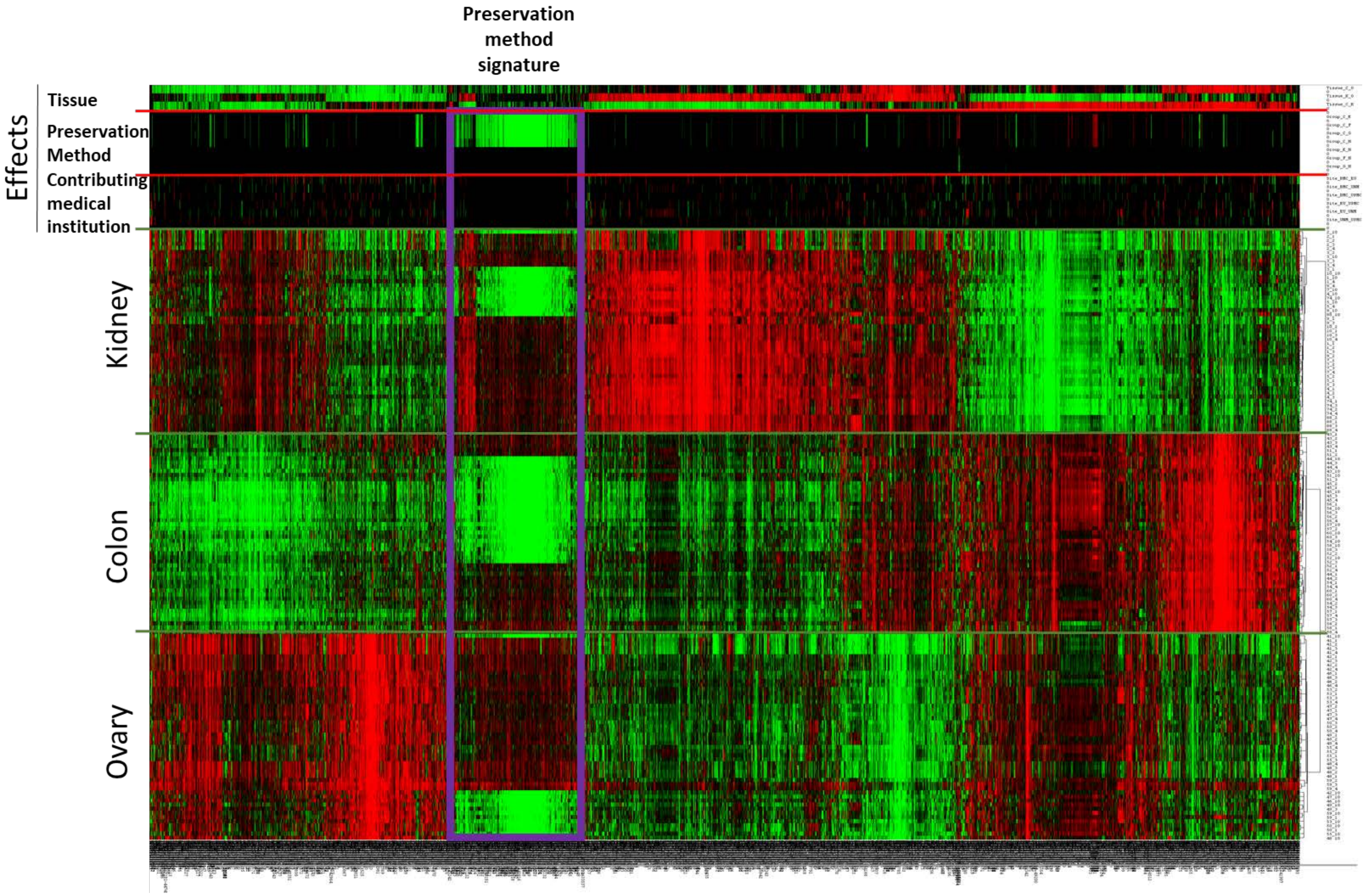

Supplementary Figure 2: Heat map of more than 3300 genes in 148 specimens, modeled by the linear model, that were significantly affected by at least one factor and displayed more than a 4-fold difference. Distinct genes are in columns and samples are rows. Illustrated at the top are genes that were significantly affected by one or more contrasts related to preservation group, tissue, or contributing medical institution. Colon specimen 51-4 and kidney specimen 4-4 were omitted.

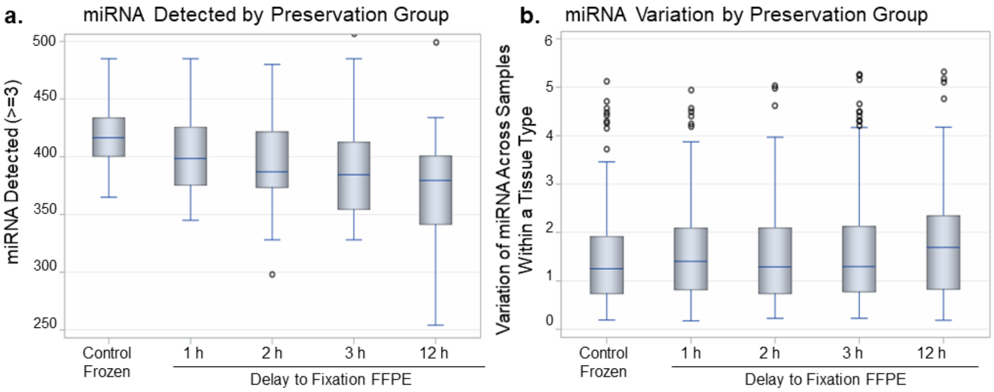

Supplemental Figure 3: The box plots show the number of miRNA detected (>=3 counts) **(a)** and the variation in >1400 individual miRNA-Seq log normalized counts **(b)** in snap-frozen specimens (n=30) and FFPE specimens fixed after 1, 2, 3 or 12 h DTF (n=30, each timepoint). The average number of miRNA detected decreased as the delay to fixation increased, and the typical standard deviation (std dev) for detected miRNA was 35% higher (1.69 vs. 1.25) in specimens subjected to a 12 h DTF compared to snap-frozen specimens. Additionally, a subset of the miRNA dramatically increased their variation as DTF increased, having a std dev in expression at the 12 h DTF that was 2-5 times the comparable value from control specimens. The upper and lower extremes of the box correspond to the first (Q1) and third (Q3) quartiles and the whiskers show the range of the data up to 1.5 times the interquartile range (Q3-Q1). Data more extreme than the range of the whiskers are graphed as individual points.

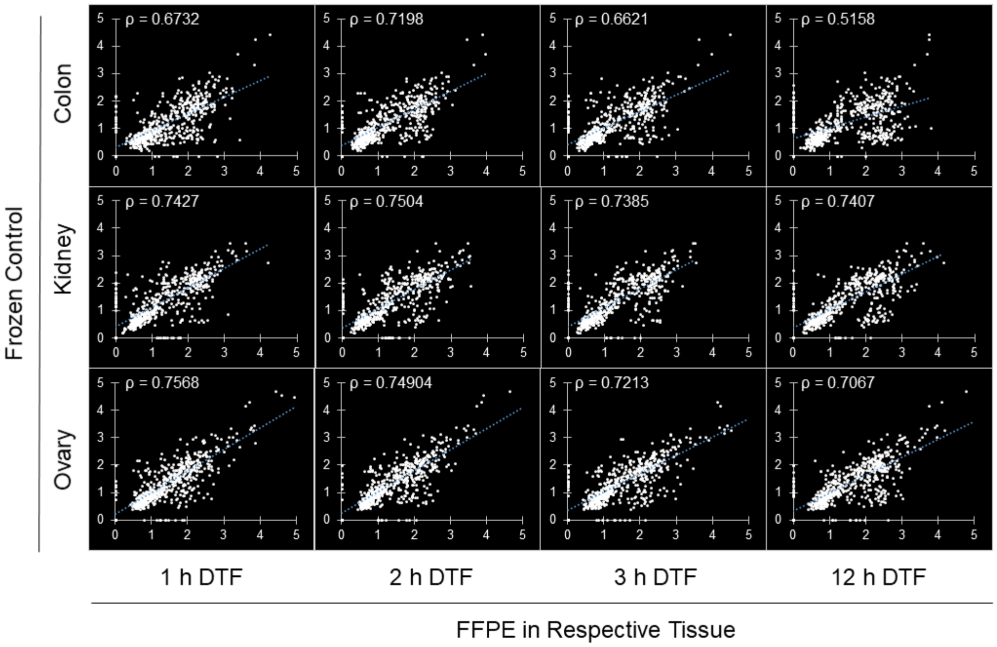

Supplemental Figure 4: Scatter plots showing standard deviation in expression of miRNA in frozen control specimens (30 for each tissue) versus the each of the corresponding DTF timepoints (30 per timepoint) in colon, kidney and ovary. Plotted values are raw std dev values. The Spearman rank correlation coefficients are displayed in the upper left corner of each plot. Increased variability is particularly evident in colon and ovarian specimens subjected to a 12 h DTF.

Supplemental Table 1: Specimen characteristics and quality control data for relevant specimens from Carithers et al. 2018[14].

SPECIMEN ID	BPV SPECIMEN ID	DTF	TISSUE TYPE	HISTOLOGIC TYPE BASED ON QC SLIDE	% TUMOR	RIN	DV200	GAPDH Q-SCORE	PGK Q-SCORE	*DNA KAPA Q129/Q41 RATIO*	*DNA KAPA Q305/Q41 RATIO*
**1-10**	BPV-00015-20	Frozen	KIDNEY	Clear cell renal carcinoma		9.8	79	1.0232	0.9066	0.989	1.075
**1-2**	BPV-00015-02	1 h	KIDNEY	Clear cell renal carcinoma	75-100	2.5	72	0.1482	0.1302	0.656	0.220
**1-4**	BPV-00015-04	2 h	KIDNEY	Clear cell renal carcinoma	75-100	2.4	77	0.1841	0.1443	0.697	0.239
**1-1**	BPV-00015-01	3 h	KIDNEY	Clear cell renal carcinoma	75-100	2.5	65	0.1512	0.1407	0.726	0.265
**1-3**	BPV-00015-03	12 h	KIDNEY	Clear cell renal carcinoma	75-100	1.9	71	0.3218	0.2743	0.757	0.387
**2-10**	BPV-00042-20	Frozen	KIDNEY	Clear cell renal carcinoma		3.4	67			0.972	0.899
**2-1**	BPV-00042-01	1 h	KIDNEY	Clear cell renal carcinoma	75-100	2.5	54	0.1197	0.0791	0.486	0.080
**2-3**	BPV-00042-03	2 h	KIDNEY	Clear cell renal carcinoma	75-100					0.545	0.104
**2-2**	BPV-00042-02	3 h	KIDNEY	Clear cell renal carcinoma	75-100	2.5	48	0.1048	0.0717	0.489	0.053
**2-4**	BPV-00042-04	12 h	KIDNEY	Clear cell renal carcinoma	75-100					0.710	0.200
**3-10**	BPV-00044-20	Frozen	KIDNEY	Clear cell renal carcinoma		10	88	0.2539	0.3362	0.892	0.874
**3-4**	BPV-00044-05	1 h	KIDNEY	Clear cell renal carcinoma	75-100	2.3	64	0.1308	0.1076	0.553	0.147
**3-3**	BPV-00044-04	2 h	KIDNEY	Clear cell renal carcinoma	75-100	2.5	63	0.113	0.0978	0.494	0.158
**3-2**	BPV-00044-03	3 h	KIDNEY	Clear cell renal carcinoma	75-100	2.4	61	0.148	0.1208	0.577	0.136
**3-1**	BPV-00044-02	12 h	KIDNEY	Clear cell renal carcinoma	75-100	2.6	79	0.4353	0.3968	0.704	0.421
**4-10**	BPV-00051-20	Frozen	KIDNEY	Clear cell renal carcinoma		6.7	60	0.7502	0.74	0.867	0.813
**4-1**	BPV-00051-02	1 h	KIDNEY	Clear cell renal carcinoma	75-100	2.4	60	0.1403	0.1263	0.507	0.183
**4-2**	BPV-00051-03	2 h	KIDNEY	Clear cell renal carcinoma	75-100	2.5	60	0.128	0.103	0.568	0.202
**4-3**	BPV-00051-04	3 h	KIDNEY	Clear cell renal carcinoma	75-100	2.4	34	0.0785	0.0662	0.372	0.078
**4-4**	BPV-00051-05	12 h	KIDNEY	Clear cell renal carcinoma	75-100	2	39	0.2226	0.1635	0.630	0.352
**5-10**	BPV-00088-20	Frozen	KIDNEY	Clear cell renal carcinoma		9.5	31	0.857	0.8765	0.967	0.831
**5-1**	BPV-00088-01	1 h	KIDNEY	Clear cell renal carcinoma	75-100	3.1	56	0.1581	0.1263	0.561	0.143
**5-4**	BPV-00088-04	2 h	KIDNEY	Clear cell renal carcinoma	75-100	2.7	58	0.1737	0.134	0.577	0.186
**5-3**	BPV-00088-03	3 h	KIDNEY	Clear cell renal carcinoma	75-100	3.3	56	0.1444	0.1021	0.576	0.151
**5-2**	BPV-00088-02	12 h	KIDNEY	Clear cell renal carcinoma	75-100	2.4	68	0.4451	0.3591	0.703	0.454
**7-10**	BPV-00099-22	Frozen	KIDNEY	Clear cell renal carcinoma		9.8	65	0.7841	0.9939	0.889	0.765
**7-2**	BPV-00099-02	1 h	KIDNEY	Clear cell renal carcinoma	75-100	2.6	60			0.485	0.177
**7-3**	BPV-00099-03	2 h	KIDNEY	Clear cell renal carcinoma	75-100	2.6	71	0.1572	0.1281	0.536	0.162
**7-4**	BPV-00099-04	3 h	KIDNEY	Clear cell renal carcinoma	75-100	2.4	61	0.1534	0.1204	0.586	0.126
**7-1**	BPV-00099-01	12 h	KIDNEY	Clear cell renal carcinoma	75-100	3	75	0.4394	0.361	0.623	0.333
**9-10**	BPV-00142-20	Frozen	KIDNEY	Clear cell renal carcinoma		7.5	65	0.7939	0.9631	0.933	0.865
**9-4**	BPV-00142-04	1 h	KIDNEY	Clear cell renal carcinoma	50-74	3.1	70	0.1305	0.1242	0.578	0.171
**9-1**	BPV-00142-01	2 h	KIDNEY	Clear cell renal carcinoma	75-100	3.6	74	0.1614	0.1427	0.484	0.152
**9-2**	BPV-00142-02	3 h	KIDNEY	Clear cell renal carcinoma	75-100	2.4	70	0.1482	0.1536	0.496	0.170
**9-3**	BPV-00142-03	12 h	KIDNEY	Clear cell renal carcinoma	75-100	3.1	81	0.4722	0.4059	0.629	0.436
**10-10**	BPV-00245-20	Frozen	KIDNEY	Clear cell renal carcinoma		8.7	72	0.6225	0.7769		

SPECIMEN ID	BPV SPECIMEN ID	DTF	TISSUE TYPE	HISTOLOGIC TYPE BASED ON QC SLIDE	% TUMOR	RIN	DV200	GAPDH Q-SCORE	PGK Q-SCORE	*DNA KAPA Q129/Q41 RATIO*	*DNA KAPA Q305/Q41 RATIO*
**10-1**	BPV-00245-01	1 h	KIDNEY	Clear cell renal carcinoma	75-100	2.3	56	0.1606	0.1458		
**10-2**	BPV-00245-02	2 h	KIDNEY	Clear cell renal carcinoma	75-100	2.4	69	0.143	0.1469		
**10-3**	BPV-00245-03	3 h	KIDNEY	Clear cell renal carcinoma	75-100	3.1	67	0.1542	0.1387		
**10-4**	BPV-00245-04	3 h	KIDNEY	Clear cell renal carcinoma	75-100	2.5	73	0.3578	0.3157		
**41-10**	BPV-00001-20	Frozen	OVARY	Serous carcinoma		8	59	0.7758	0.7059	0.832	0.905
**41-3**	BPV-00001-08	1 h	OVARY	Serous carcinoma	75-100	2.4	75	0.1945	0.1918	0.689	0.179
**41-2**	BPV-00001-06	2 h	OVARY	Serous carcinoma	75-100	2.3	68	0	0	0.643	0.099
**41-1**	BPV-00001-05	3 h	OVARY	Serous carcinoma	75-100	2.4	70	0.1012	0.0924	0.644	0.155
**41-4**	BPV-00001-09	12 h	OVARY	Serous carcinoma	75-100	2.1	70	0.4509	0.452	0.781	0.520
**42-10**	BPV-00011-20	Frozen	OVARY	Serous carcinoma		9.2	49	0.8451	0.8684	0.834	0.931
**42-4**	BPV-00011-08	1 h	OVARY	Serous carcinoma	75-100	2.4	70	0.1901	0.1612	0.595	0.174
**42-1**	BPV-00011-05	2 h	OVARY	Serous carcinoma	75-100	2.2	58	0.1322	0.1269	0.575	0.178
**42-2**	BPV-00011-06	3 h	OVARY	Serous carcinoma	75-100	2.1	68	0.1683	0.1417	0.742	0.157
**42-3**	BPV-00011-07	12 h	OVARY	Serous carcinoma	75-100	2.2	38	0.2735	0.2554	0.580	0.305
**43-10**	BPV-00032-20	Frozen	COLON	Adenocarcinoma of colon		7.8	57	0.9986	0.8077	1.005	0.978
**43-3**	BPV-00032-08	1 h	COLON	Adenocarcinoma of colon	50-74	2.7	71	0.0745	0.0861	0.604	0.146
**43-1**	BPV-00032-06	2 h	COLON	Adenocarcinoma of colon	75-100	2.8	79	0.1141	0.1137	0.652	0.155
**43-2**	BPV-00032-07	3 h	COLON	Adenocarcinoma of colon	50-74	2.4	69	0.0708	0.0736	0.557	0.142
**43-4**	BPV-00032-09	12 h	COLON	Adenocarcinoma of colon	75-100	2.3	50	0.0862	0.1094	0.745	0.306
**44-10**	BPV-00034-20	Frozen	COLON	Adenocarcinoma of colon		9.3	85	0.9804	0.8017	0.804	0.794
**44-2**	BPV-00034-02	1 h	COLON	Adenocarcinoma of colon	50-74	2.3	70	0.0615	0.074	0.663	0.190
**44-4**	BPV-00034-04	2 h	COLON	Adenocarcinoma of colon	75-100	2.3	73	0.1105	0.1054	0.694	0.154
**44-1**	BPV-00034-01	3 h	COLON	Adenocarcinoma of colon	50-74	2.3	54	0.019	0.0226	0.744	0.209
**44-3**	BPV-00034-03	12 h	COLON	Adenocarcinoma of colon	75-100	2.1	79	0.2961	0.3139	0.702	0.381
**45-10**	BPV-00070-20	Frozen	COLON	Other		9.6	86	1.0554	0.8444	0.863	0.885
**45-1**	BPV-00070-01	1 h	COLON	Other	75-100	2.3	86	0.25	0.2227	0.635	0.266
**45-2**	BPV-00070-02	2 h	COLON	Other	75-100	2.2	90	0.4195	0	0.684	0.270
**45-3**	BPV-00070-03	3 h	COLON	Other	75-100	2.1	88	0.0368	0.042	0.659	0.279
**45-4**	BPV-00070-04	12 h	COLON	Other	75-100	2.4	61	0	0	0.709	0.378
**46-10**	BPV-00083-20	Frozen	OVARY	Serous carcinoma		9.6	84	1.0679	1.0325	0.916	0.867
**46-1**	BPV-00083-01	1 h	OVARY	Serous carcinoma	75-100	3.7	75	0.1182	0.1271	0.547	0.200
**46-4**	BPV-00083-04	2 h	OVARY	Serous carcinoma	75-100	2.3	76	0.1501	0.1674	0.629	0.172
**46-2**	BPV-00083-02	3 h	OVARY	Serous carcinoma	50-74	2.5	68	0.1296	0.1495	0.540	0.174
**46-3**	BPV-00083-03	12 h	OVARY	Serous carcinoma	75-100	2.1	69	0.3081	0.3337	0.737	0.454
**47-10**	BPV-00090-20	Frozen	OVARY	Serous cystadenocarcinoma		9.7	77	0.8827	0.8494	1.082	0.933
**47-1**	BPV-00090-01	1 h	OVARY	Serous cystadenocarcinoma	75-100	2.3	77	0.3981	0.3868	0.705	0.302
**47-4**	BPV-00090-04	2 h	OVARY	Serous cystadenocarcinoma	75-100	2.1	72	0.3551	0.3642	0.713	0.327
**47-3**	BPV-00090-03	3 h	OVARY	Serous cystadenocarcinoma	75-100	2.3	83	0.4568	0.3661	0.707	0.359
**47-2**	BPV-00090-02	12 h	OVARY	Serous cystadenocarcinoma	75-100	2.4	79	0.3585	0.3285	0.668	0.280

SPECIMEN ID	BPV SPECIMEN ID	DTF	TISSUE TYPE	HISTOLOGIC TYPE BASED ON QC SLIDE	% TUMOR	RIN	DV200	GAPDH Q-SCORE	PGK Q-SCORE	*DNA KAPA Q129/Q41 RATIO*	*DNA KAPA Q305/Q41 RATIO*
**48-10**	BPV-00097-20	Frozen	OVARY	Serous carcinoma		9.6	78	0.8472	0.8942	0.951	0.920
**48-3**	BPV-00097-03	1 h	OVARY	Serous carcinoma	75-100	2.3	74	0.1663	0.1473	0.535	0.329
**48-4**	BPV-00097-04	2 h	OVARY	Serous carcinoma	75-100	2.5	74	0.2095	0.1706	0.594	0.233
**48-1**	BPV-00097-01	3 h	OVARY	Serous carcinoma	75-100	2.7	76	0.1606	0.1197	0.564	0.127
**48-2**	BPV-00097-02	12 h	OVARY	Serous carcinoma	75-100	2.5	78	0.3283	0.2867	0.663	0.336
**49-10**	BPV-00108-20	Frozen	OVARY	Serous carcinoma		9.4	58	1.0031	0.9379	1.208	1.197
**49-3**	BPV-00108-03	1 h	OVARY	Serous carcinoma	50-74	2.4	55			0.470	0.111
**49-4**	BPV-00108-04	2 h	OVARY	Serous carcinoma	75-100	2.5	69	0.0966	0.0938	0.390	0.046
**49-2**	BPV-00108-02	3 h	OVARY	Serous carcinoma	75-100	2.6	72	0.1033	0.1056	0.460	0.101
**49-1**	BPV-00108-01	12 h	OVARY	Serous carcinoma	75-100	2.8	84	0.3338	0.2928	0.629	0.282
**50-10**	BPV-00148-20	Frozen	OVARY	Serous carcinoma		7.6	62	0.9511	0.767	1.075	1.055
**50-4**	BPV-00148-04	1 h	OVARY	Serous carcinoma	75-100	2.4	71	0.1203	0.1358	0.526	0.175
**50-2**	BPV-00148-02	2 h	OVARY	Serous carcinoma	75-100	2.4	74	0.1372	0.1289	0.484	0.158
**50-3**	BPV-00148-03	3 h	OVARY	Serous carcinoma	75-100	2.5	69	0.1888	0.1746	0.511	0.185
**50-1**	BPV-00148-01	12 h	OVARY	Serous carcinoma	75-100	2.2	66	0.2511	0.2531	0.720	0.486
**51-10**	BPV-00156-20	Frozen	COLON	Carcinoma of colon, mucinous		8.8	60	0.7144	0.6878	1.157	0.993
**51-4**	BPV-00156-04	1 h	COLON	Carcinoma of colon, mucinous	25-49	2.5	57	0.0029	0	0.498	0.132
**51-2**	BPV-00156-02	2 h	COLON	Carcinoma of colon, mucinous	50-74	2.4	55	0.1403	0.1226	0.626	0.169
**51-1**	BPV-00156-01	3 h	COLON	Carcinoma of colon, mucinous	50-74	2.3	70			0.619	0.187
**51-3**	BPV-00156-03	12 h	COLON	Carcinoma of colon, mucinous	75-100	2.2	88	0.2807	0.2469	0.699	0.329
**52-10**	BPV-00184-20	Frozen	COLON	Carcinoma of colon, mucinous		7.8	76	1.1277	0.9362	1.032	0.855
**52-1**	BPV-00184-01	1 h	COLON	Carcinoma of colon, mucinous	75-100	2.4	56	0.1965	0.1739	0.641	0.155
**52-2**	BPV-00184-02	2 h	COLON	Carcinoma of colon, mucinous	50-74	2.3	60	0.1724	0.1535	0.723	0.104
**52-3**	BPV-00184-03	3 h	COLON	Carcinoma of colon, mucinous	50-74	2.3	64	0.2255	0.175	0.613	0.152
**52-4**	BPV-00184-04	12 h	COLON	Carcinoma of colon, mucinous	50-74	2.3	67	0.4229	0.3561	0.899	0.306
**53-10**	BPV-00218-27	Frozen	OVARY	Serous carcinoma		9.2	74	0.8611	0.889	1.005	1.028
**53-3**	BPV-00218-03	1 h	OVARY	Serous carcinoma	75-100	2.5	67	0.1302	0.1544	0.467	0.083
**53-4**	BPV-00218-04	2 h	OVARY	Serous carcinoma	75-100	2.1	69	0.1739	0.1785	0.578	0.227
**53-1**	BPV-00218-01	12 h	OVARY	Serous carcinoma	50-74	2.2	76	0.3691	0.3456	0.772	0.392
**53-2**	BPV-00218-02	QC	OVARY	Serous carcinoma	75-100						
**54-10**	BPV-00220-20	Frozen	COLON	Adenocarcinoma of colon		9.3	76	0.8498	0.8418	1.164	0.909
**54-2**	BPV-00220-02	1 h	COLON	Adenocarcinoma of colon	50-74	2.3	74	0.1611	0.1403	0.570	0.068
**54-3**	BPV-00220-03	2 h	COLON	Adenocarcinoma of colon	75-100	2.5	77	0.1593	0.1483	0.627	0.084
**54-1**	BPV-00220-01	3 h	COLON	Adenocarcinoma of colon	25-49	2.1	81	0.2009	0.1798	0.566	0.112
**54-4**	BPV-00220-15	12 h	COLON	Adenocarcinoma of colon	25-49	2.2	78	0.3687	0.3453	0.865	0.423
**55-10**	BPV-00242-20	Frozen	OVARY	Serous carcinoma		9.1	76	0.7978	0.7848	1.245	1.085
**55-1**	BPV-00242-02	1 h	OVARY	Serous carcinoma	75-100	2.2	77	0.137	0.1322	0.881	0.272
**55-2**	BPV-00242-03	2 h	OVARY	Serous carcinoma	75-100	2.5	76	0.1381	0.1393	0.801	0.199
**55-3**	BPV-00242-04	3 h	OVARY	Serous carcinoma	75-100	2.1	78	0.1435	0.1284	0.712	0.213

SPECIMEN ID	BPV SPECIMEN ID	DTF	TISSUE TYPE	HISTOLOGIC TYPE BASED ON QC SLIDE	% TUMOR	RIN	DV200	GAPDH Q-SCORE	PGK Q-SCORE	*DNA KAPA Q129/Q41 RATIO*	*DNA KAPA Q305/Q41 RATIO*
**55-4**	BPV-00242-05	12 h	OVARY	Serous carcinoma	75-100	2.7	85	0.4272	0.3529	1.018	0.591
**56-10**	BPV-00255-21	Frozen	COLON	Adenocarcinoma of colon		9.5	87	1.0339	0.9382	1.126	0.974
**56-3**	BPV-00255-03	1 h	COLON	Adenocarcinoma of colon	75-100	2.4	60	0.2753	0.3148	0.664	0.339
**56-4**	BPV-00255-04	2 h	COLON	Adenocarcinoma of colon	75-100	2.4	62	0.1208	0.1256	0.517	0.182
**56-1**	BPV-00255-01	3 h	COLON	Adenocarcinoma of colon	75-100	2.4	59	0.1357	0.1442	0.446	0.109
**56-2**	BPV-00255-02	12 h	COLON	Adenocarcinoma of colon	75-100	2.4	55	0.1	0.1123	0.488	0.103
**57-10**	BPV-00290-20	Frozen	COLON	Adenocarcinoma of colon		9.1	91	0.8823	0.8962	0.879	0.904
**57-1**	BPV-00290-01	1 h	COLON	Adenocarcinoma of colon	75-100	2.3	76	0.1277	0.1428	0.588	0.185
**57-4**	BPV-00290-04	2 h	COLON	Adenocarcinoma of colon	75-100	2.4	65	0.1319	0.1265	0.538	0.153
**57-3**	BPV-00290-03	3 h	COLON	Adenocarcinoma of colon	75-100	2.9	78	0.1214	0.1205	0.552	0.190
**57-2**	BPV-00290-02	12 h	COLON	Adenocarcinoma of colon	75-100	2.3	75	0.183	0.1952	0.642	0.248
**58-10**	BPV-00304-20	Frozen	COLON	Adenocarcinoma of colon		8.5	77	0.9015	0.8339	0.993	1.035
**58-1**	BPV-00304-01	1 h	COLON	Adenocarcinoma of colon	75-100	2.4	59	0.0343	0.0295	0.542	0.158
**58-2**	BPV-00304-02	2 h	COLON	Adenocarcinoma of colon	75-100	2.4	68	0.0656	0.0541	0.605	0.115
**58-3**	BPV-00304-03	3 h	COLON	Adenocarcinoma of colon	50-74	2.8	74	0.1396	0.1237	0.512	0.130
**58-4**	BPV-00304-04	12 h	COLON	Adenocarcinoma of colon	50-74	2.4	74	0.3877	0.33	0.870	0.586
**59-10**	BPV-00364-20	Frozen	OVARY	Serous carcinoma		9.1	72	0.9184	0.7971	0.977	0.898
**59-1**	BPV-00364-01	1 h	OVARY	Serous carcinoma	75-100	2.4	66	0.0932	0.1113	0.603	0.224
**59-2**	BPV-00364-02	2 h	OVARY	Serous carcinoma	75-100	2.4	52	0.1363	0.0779	0.682	0.251
**59-3**	BPV-00364-03	3 h	OVARY	Serous carcinoma	75-100	2.4	40	0.0575	0.0152	0.682	0.209
**59-4**	BPV-00364-04	12 h	OVARY	Serous carcinoma	75-100	2.5	42	0.1553	0.0599	0.712	0.351
**60-10**	BPV-00410-20	Frozen	COLON	Adenocarcinoma of colon		9.5	64	0.8194	0.8346	0.926	0.910
**60-2**	BPV-00410-02	1 h	COLON	Adenocarcinoma of colon	75-100	3.9	80	0.1204	0.0966	0.676	0.205
**60-4**	BPV-00410-16	2 h	COLON	Adenocarcinoma of colon	50-74	3.3	80	0.1507	0.1533	0.596	0.150
**60-1**	BPV-00410-01	3 h	COLON	Adenocarcinoma of colon	75-100	3.9	74	0.1488	0.1114	0.587	0.172
**60-3**	BPV-00410-15	12 h	COLON	Adenocarcinoma of colon	50-74	2.9	83	0.4171	0.4022	0.767	0.528
**74-10**	BPV-00301-20	Frozen	KIDNEY	Clear cell renal carcinoma		9.6	79	0.7735	0.8036		
**74-1**	BPV-00301-01	1 h	KIDNEY	Clear cell renal carcinoma	50-74	2.3	66	0.1915	0.1892		
**74-2**	BPV-00301-02	2 h	KIDNEY	Clear cell renal carcinoma	75-100	2.2	71	0.2118	0.1869		
**74-3**	BPV-00301-03	3 h	KIDNEY	Clear cell renal carcinoma	75-100	2.5	71	0.1871	0.198		
**74-4**	BPV-00301-04	12 h	KIDNEY	Clear cell renal carcinoma	75-100	2.2	74	0.3172	0.2877		
**88-10**	BPV-00003-20	Frozen	KIDNEY	Clear cell renal carcinoma		9.1	87	0.8814	0.8361	1.026	0.914
**88-1**	BPV-00003-05	1 h	KIDNEY	Clear cell renal carcinoma	50-74	2.8	60	0.0076	0	0.403	0.053
**88-2**	BPV-00003-06	2 h	KIDNEY	Clear cell renal carcinoma	50-74	2.3	59	0.0058	0	0.420	0.036
**88-3**	BPV-00003-07	3 h	KIDNEY	Clear cell renal carcinoma	50-74	2.1	58	0.0147	0	0.480	0.055
**88-4**	BPV-00003-08	12 h	KIDNEY	Clear cell renal carcinoma	50-74	2.3	67	0.2802	0.2954	0.502	0.163

Supplementary Table 2: Basic RNA-Seq summary data organized by sample. The complete dataset is available through dbGaP (phs001639).

Specimen ID	Tissue	Pres type	DTF	Genes detected	Genes above 0 counts	Percent of Genes found	Back-ground Detection threshold	Median count	Total Clusters (Mil)	Total M Clusters (post-clipping)	Clip Pct	%rRNA	Trans-script-ome mapped
1-10	Kidney	Frozen	Ctl	17441	22149	50.6	9.3	257	56.4	54.8	2.76	1.31	63.9
1-2	Kidney	FFPE	1h	15722	23263	45.6	18.8	127	56.5	55.2	2.38	1.82	31.3
1-4	Kidney	FFPE	2h	15247	21914	44.2	16.8	136	53.9	52.7	2.2	1.67	31.1
1-1	Kidney	FFPE	3h	15674	22973	45.4	18.2	124	57.8	56.3	2.53	1.74	29.9
1-3	Kidney	FFPE	12h	15830	23232	45.9	17.2	120	55.9	54.6	2.26	1.75	30.4
2-10	Kidney	Frozen	Ctl	15980	21219	46.3	11.7	209	53.1	51.6	2.74	1.03	48.5
2-1	Kidney	FFPE	1h	15393	22134	44.6	14.8	112	57.1	55.8	2.32	1.22	28.5
2-3	Kidney	FFPE	2h	14912	22340	43.2	17.6	108	54.7	53.3	2.49	1.40	28.6
2-2	Kidney	FFPE	3h	15204	21851	44.1	15.3	119	58.3	56.8	2.59	1.50	31.4
2-4	Kidney	FFPE	12h	15124	22155	43.8	16.7	121	56.3	55.0	2.46	2.32	32.4
3-1	Kidney	Frozen	Ctl	15102	21766	43.8	17.9	149	56.7	55.2	2.67	2.61	32.6
3-1R2	Kidney	Frozen	Ctl	15312	21836	44.4	18.1	164	58.1	55.4	4.6	1.73	34.5
3-4	Kidney	FFPE	1h	15146	23002	43.9	19.1	100	55.5	54.2	2.44	3.25	26.0
3-3	Kidney	FFPE	2h	15270	22814	44.3	18.2	102	53.9	52.1	3.36	3.97	28.2
3-2	Kidney	FFPE	3h	15201	22658	44.1	18.8	116	55.5	54.1	2.63	3.04	30.3
3-10	Kidney	FFPE	12h	15886	23617	46.1	21.5	135	59.9	58.7	2.09	2.12	30.8
3-10R2	Kidney	FFPE	12h	15665	23892	45.4	26.2	150	71.2	68.2	4.22	2.54	31.9
4-10	Kidney	Frozen	Ctl	16805	21624	48.7	9.7	245	53.1	51.4	3.26	0.87	60.8
4-1	Kidney	FFPE	1h	15292	22932	44.3	19.1	115	56.9	55.6	2.27	1.67	27.6
4-2	Kidney	FFPE	2h	15014	22714	43.5	19.4	112	56.8	55.5	2.35	1.72	27.1
4-3	Kidney	FFPE	3h	14989	23745	43.5	17.9	82	56.9	55.4	2.64	11.35	32.2
4-4	Kidney	FFPE	12h	7938	29367	23.0	39.8	32	56.7	55.5	2.27	0.41	8.0
5-10	Kidney	Frozen	Ctl	16657	21103	48.3	7.5	189	53.1	51.5	3.11	1.00	66.2
5-1	Kidney	FFPE	1h	14766	22805	42.8	20.0	100	52.0	50.8	2.46	1.06	27.0
5-4	Kidney	FFPE	2h	14409	20887	41.8	17.1	137	51.7	50.6	2.2	1.59	32.9
5-3	Kidney	FFPE	3h	14989	22766	43.5	20.4	113	57.6	56.1	2.56	1.08	28.9
5-2	Kidney	FFPE	12h	15666	22821	45.4	15.4	115	54.3	52.8	2.77	2.19	32.9
7-10	Kidney	Frozen	Ctl	18193	21696	52.7	4.9	255	50.6	49.4	2.42	0.76	66.5
7-2	Kidney	FFPE	1h	15908	23196	46.1	17.4	125	56.0	54.9	1.93	1.55	30.9
7-3	Kidney	FFPE	2h	15494	22527	44.9	15.7	115	52.1	51.0	2.01	1.15	28.6
7-4	Kidney	FFPE	3h	15769	22207	45.7	14.1	124	53.4	52.4	1.76	1.47	32.2
7-1	Kidney	FFPE	12h	16241	23491	47.1	16.1	127	54.0	52.8	2.19	1.69	31.7
9-10	Kidney	Frozen	Ctl	17174	21877	49.8	9.6	203	57.6	55.8	3.18	7.20	56.9
9-4	Kidney	FFPE	1h	14383	21534	41.7	24.5	159	60.0	58.5	2.44	1.75	31.0
9-1	Kidney	FFPE	2h	14466	22087	41.9	20.5	120	53.0	52.0	2.01	1.06	30.2
9-2	Kidney	FFPE	3h	15373	23238	44.6	19.0	113	52.2	51.1	2.15	1.72	30.3

Specimen ID	Tissue	Pres type	DTF	Genes detected	Genes above 0 counts	Percent of Genes found	Back-ground Detection threshold	Median count	Total Clusters (Mil)	Total M Clusters (post-clipping)	Clip Pct	%rRNA	Trans-script-ome mapped
9-3	Kidney	FFPE	12h	14999	22713	43.5	21.5	138	61.9	60.5	2.37	1.92	32.3
10-10	Kidney	Frozen	Ctl	16933	21970	49.1	11.5	273	63.7	61.8	2.97	2.36	59.0
10-1	Kidney	FFPE	1h	15302	23650	44.4	19.4	107	56.1	54.7	2.52	1.58	29.2
10-2	Kidney	FFPE	2h	15373	23203	44.6	20.4	123	59.2	57.5	2.88	1.76	29.0
10-3	Kidney	FFPE	3h	15364	22990	44.5	19.8	128	61.5	59.8	2.67	1.59	29.9
10-4	Kidney	FFPE	12h	16014	23431	46.4	18.6	144	67.7	66.1	2.34	2.30	33.9
41-10	Ovary	Frozen	Ctl	14940	21802	43.3	18.5	169	60.3	58.6	2.91	0.80	46.8
41-3	Ovary	FFPE	1h	12425	23342	36.0	37.9	89	62.8	61.2	2.57	1.03	20.3
41-2	Ovary	FFPE	2h	12196	23078	35.4	39.1	90	62.6	61.2	2.31	1.01	19.9
41-1	Ovary	FFPE	3h	12443	23143	36.1	36.8	91	64.0	62.7	2.13	1.71	21.4
41-4	Ovary	FFPE	12h	13393	23533	38.8	33.4	100	57.4	55.8	2.78	1.42	24.2
42-10	Ovary	Frozen	Ctl	17087	23128	49.5	15.1	244	61.5	59.7	2.93	1.54	56.5
42-4	Ovary	FFPE	1h	13668	24149	39.6	35.0	102	61.2	59.6	2.53	1.05	23.8
42-1	Ovary	FFPE	2h	13516	23686	39.2	33.5	101	60.9	59.4	2.52	1.18	24.2
42-2	Ovary	FFPE	3h	13868	24125	40.2	34.6	109	65.0	63.7	2.01	1.04	24.6
42-3	Ovary	FFPE	12h	14037	24007	40.7	32.1	107	61.3	59.5	2.87	1.20	25.9
43-10	Colon	Frozen	Ctl	16765	21850	48.6	8.4	168	54.2	52.6	2.99	1.98	58.2
43-3	Colon	FFPE	1h	15498	23648	44.9	22.3	124	59.3	57.8	2.59	2.19	28.0
43-1	Colon	FFPE	2h	14944	23504	43.3	24.3	120	63.1	61.8	2.15	1.31	27.0
43-2	Colon	FFPE	3h	15861	23331	46.0	19.0	130	57.8	56.3	2.53	3.48	30.6
43-4	Colon	FFPE	12h	16696	23737	48.4	17.0	139	57.7	55.9	3.14	7.34	36.5
44-10	Colon	Frozen	Ctl	18409	22248	53.4	5.6	265	58.7	56.7	3.55	2.72	63.5
44-2	Colon	FFPE	1h	16671	23138	48.3	12.3	122	57.2	55.2	3.45	2.90	33.2
44-4	Colon	FFPE	2h	16007	21316	46.4	10.2	162	58.4	57.1	2.15	2.41	36.6
44-1	Colon	FFPE	3h	17274	21681	50.1	8.7	151	59.8	57.0	4.74	10.70	40.5
44-3	Colon	FFPE	12h	16177	21675	46.9	10.4	178	62.9	60.7	3.47	3.99	40.3
45-10	Colon	Frozen	Ctl	16822	21876	48.8	9.0	256	67.2	64.8	3.51	2.47	65.3
45-1	Colon	FFPE	1h	14518	21278	42.1	17.7	162	67.3	65.3	2.96	1.92	31.8
45-2	Colon	FFPE	2h	14215	21410	41.2	22.4	172	70.2	68.4	2.48	2.19	33.0
45-3	Colon	FFPE	3h	14351	21583	41.6	19.8	154	65.9	64.1	2.81	2.11	32.6
45-4	Colon	FFPE	12h	15151	21273	43.9	12.3	149	54.8	53.7	2.16	2.45	35.2
46-10	Ovary	Frozen	Ctl	17538	22853	50.8	10.5	233	55.6	54.4	2.1	1.38	58.6
46-1	Ovary	FFPE	1h	14803	23517	42.9	24.3	112	58.8	57.7	1.86	1.19	27.1
46-4	Ovary	FFPE	2h	14750	23934	42.8	23.4	96	53.5	52.6	1.84	1.44	25.9
46-2	Ovary	FFPE	3h	15608	23615	45.3	17.9	101	51.5	50.4	2.05	1.53	28.0
46-3	Ovary	FFPE	12h	16406	23875	47.6	16.2	119	51.8	50.9	1.79	3.37	34.4
47-10	Ovary	Frozen	Ctl	17526	22487	50.8	8.5	221	55.3	54.0	2.3	1.07	63.2
47-1	Ovary	FFPE	1h	15020	23609	43.5	22.0	112	54.9	53.9	1.85	1.90	27.1
47-4	Ovary	FFPE	2h	15086	23702	43.7	23.9	123	65.4	64.4	1.49	1.40	26.9

Specimen ID	Tissue	Pres type	DTF	Genes detected	Genes above 0 counts	Percent of Genes found	Back-ground Detection threshold	Median count	Total Clusters (Mil)	Total M Clusters (post-clipping)	Clip Pct	%rRNA	Trans-script-ome mapped
47-3	Ovary	FFPE	3h	15337	23667	44.5	21.1	122	62.0	60.9	1.66	1.27	27.7
47-2	Ovary	FFPE	12h	15191	23725	44.0	22.6	124	65.8	64.5	1.96	1.66	28.7
48-10	Ovary	Frozen	Ctl	16868	22133	48.9	10.9	204	52.4	51.6	1.6	1.29	51.7
48-3	Ovary	FFPE	1h	14747	22911	42.8	20.0	96	52.1	51.3	1.6	1.48	22.9
48-4	Ovary	FFPE	2h	14802	22709	42.9	15.8	78	50.6	49.8	1.71	19.73	36.9
48-1	Ovary	FFPE	3h	15076	23263	43.7	20.6	103	59.6	58.5	1.94	1.29	22.6
48-2	Ovary	FFPE	12h	15273	23219	44.3	19.3	104	52.1	51.3	1.56	2.46	25.7
49-10	Ovary	Frozen	Ctl	17536	22177	50.8	7.4	228	51.2	50.1	2.28	1.07	65.8
49-3	Ovary	FFPE	1h	14336	21934	41.6	20.3	115	51.7	50.9	1.66	1.43	26.1
49-4	Ovary	FFPE	2h	14428	22882	41.8	21.1	94	51.9	50.9	1.98	1.18	25.1
49-2	Ovary	FFPE	3h	14864	23083	43.1	20.3	105	56.4	55.3	2.02	2.11	29.7
49-1	Ovary	FFPE	12h	14927	23459	43.3	21.5	110	52.5	51.6	1.86	1.55	30.0
50-10	Ovary	Frozen	Ctl	15516	22186	45.0	17.7	167	53.6	52.7	1.64	0.41	42.0
50-4	Ovary	FFPE	1h	14482	23333	42.0	24.8	106	54.6	53.6	1.87	1.93	26.2
50-2	Ovary	FFPE	2h	14679	23278	42.6	21.8	101	52.6	51.6	1.85	1.23	25.1
50-3	Ovary	FFPE	3h	14357	23336	41.6	25.7	108	57.9	56.9	1.72	1.14	24.9
50-1	Ovary	FFPE	12h	14906	21911	43.2	18.5	138	51.8	50.8	1.93	1.96	30.9
51-10	Colon	Frozen	Ctl	17301	22397	50.2	8.4	178	50.9	49.7	2.35	2.71	58.8
51-4	Colon	FFPE	1h	11661	13002	33.8	3.0	45	54.1	36.3	32.88	32.26	3.4
51-4R2	Colon	FFPE	1h	11157	22131	32.3	27.9	61	66.9	58.2	13.03	33.72	50.1
51-2	Colon	FFPE	2h	16229	22882	47.1	12.8	121	51.0	49.7	2.44	3.25	32.4
51-1	Colon	FFPE	3h	15771	24822	45.7	17.5	92	52.1	51.1	1.88	1.93	29.2
51-3	Colon	FFPE	12h	15962	21826	46.3	11.8	159	54.6	53.3	2.42	4.16	37.5
52-10	Colon	Frozen	Ctl	17224	22183	49.9	8.1	177	56.7	55.0	3.04	1.02	51.5
52-1	Colon	FFPE	1h	16230	22424	47.1	11.6	119	51.7	50.4	2.35	1.14	29.7
52-2	Colon	FFPE	2h	15726	21475	45.6	13.2	165	59.9	58.1	3	1.43	31.0
52-3	Colon	FFPE	3h	15887	23232	46.1	28.3	260	121.3	117.6	3.09	2.17	32.1
52-4	Colon	FFPE	12h	17024	22992	49.4	10.2	127	58.8	57.0	3	15.44	45.8
53-10	Ovary	Frozen	Ctl	16739	23026	48.5	14.6	268	98.7	93.8	4.95	1.13	60.0
53-2	Ovary	FFPE	1h	15072	23161	43.7	19.4	104	60.2	58.7	2.4	1.22	23.6
53-3	Ovary	FFPE	2h	15256	23235	44.2	20.5	119	61.1	59.4	2.73	1.72	26.9
53-4	Ovary	FFPE	3h	15207	22622	44.1	17.5	112	57.3	55.8	2.74	1.29	27.5
53-1	Ovary	FFPE	12h	15673	23395	45.4	15.8	106	56.4	55.0	2.61	10.62	37.9
54-10	Colon	Frozen	Ctl	16765	22444	48.6	11.9	253	82.8	77.7	6.08	1.24	59.6
54-2	Colon	FFPE	1h	15355	23000	44.5	19.5	124	59.7	57.4	3.82	1.54	26.7
54-3	Colon	FFPE	2h	15077	24131	43.7	34.6	178	102.1	96.7	5.25	2.24	29.7
54-1	Colon	FFPE	3h	15535	22932	45.0	20.4	137	61.1	59.5	2.62	1.26	28.2
54-4	Colon	FFPE	12h	16924	23629	49.1	13.4	137	55.9	54.6	2.47	3.61	35.9
55-10	Ovary	Frozen	Ctl	16512	21641	47.9	9.0	215	73.3	68.1	7.14	2.12	56.3

Specimen ID	Tissue	Pres type	DTF	Genes detected	Genes above 0 counts	Percent of Genes found	Back-ground Detection threshold	Median count	Total Clusters (Mil)	Total M Clusters (post-clipping)	Clip Pct	%rRNA	Trans-script-ome mapped
55-1	Ovary	FFPE	1h	15655	23202	45.4	21.2	155	83.3	79.2	4.91	1.76	28.2
55-2	Ovary	FFPE	2h	15655	22296	45.4	16.3	142	62.4	60.7	2.68	1.70	29.8
55-3	Ovary	FFPE	3h	15485	23117	44.9	22.5	154	89.6	84.4	5.76	1.55	27.3
55-4	Ovary	FFPE	12h	16098	22522	46.7	13.6	143	60.2	58.2	3.39	2.05	33.5
56-10	Colon	Frozen	Ctl	17152	21610	49.7	6.5	230	52.6	50.8	3.25	1.39	58.7
56-3	Colon	FFPE	1h	16368	20865	47.5	7.1	186	56.4	54.7	3.01	3.67	40.3
56-4	Colon	FFPE	2h	15707	20827	45.5	10.1	151	55.3	53.6	3.04	1.69	31.6
56-1	Colon	FFPE	3h	15543	20734	45.1	10.4	170	58.1	56.5	2.82	2.09	33.1
56-2	Colon	FFPE	12h	15188	20157	44.0	10.3	166	57.2	55.6	2.82	1.65	31.4
57-10	Colon	Frozen	Ctl	16351	21448	47.4	10.0	231	57.6	55.6	3.53	0.90	57.2
57-1	Colon	FFPE	1h	15675	22367	45.4	15.1	131	57.7	55.9	3.16	1.91	31.0
57-4	Colon	FFPE	2h	15476	22012	44.9	14.2	131	57.6	55.9	2.9	4.95	35.6
57-3	Colon	FFPE	3h	15408	22344	44.7	15.3	125	54.0	52.3	3.13	1.77	32.7
57-2	Colon	FFPE	12h	15410	20887	44.7	11.7	151	55.1	53.4	3.11	3.59	33.6
58-10	Colon	Frozen	Ctl	16467	21702	47.7	9.6	172	53.5	51.6	3.41	3.03	60.4
58-1	Colon	FFPE	1h	14441	21574	41.9	21.7	120	56.0	54.0	3.55	3.54	26.1
58-2	Colon	FFPE	2h	14679	22431	42.6	23.6	116	56.6	54.8	3.11	4.03	27.1
58-3	Colon	FFPE	3h	13824	21116	40.1	21.8	128	53.0	51.7	2.41	2.06	29.1
58-4	Colon	FFPE	12h	15692	23414	45.5	19.2	125	55.2	53.9	2.51	5.18	34.7
59-10	Ovary	Frozen	Ctl	17425	22017	50.5	7.4	196	50.3	48.8	2.96	1.18	59.3
59-1	Ovary	FFPE	1h	14709	21779	42.6	17.6	116	51.9	50.5	2.54	1.76	26.3
59-2	Ovary	FFPE	2h	14064	25638	40.8	26.3	72	54.3	53.0	2.37	1.21	23.3
59-3	Ovary	FFPE	3h	10001	28093	29.0	34.9	38	53.2	51.6	3.14	2.56	13.5
59-4	Ovary	FFPE	12h	12956	28371	37.6	29.7	45	52.7	51.1	3.02	1.90	18.0
60-10	Colon	Frozen	Ctl	17720	22102	51.4	7.1	250	53.0	51.7	2.38	1.16	62.6
60-2	Colon	FFPE	1h	16117	22506	46.7	12.6	119	52.2	50.9	2.56	0.93	28.8
60-4	Colon	FFPE	2h	16417	23292	47.6	16.1	139	58.6	57.4	2.04	1.70	30.5
60-1	Colon	FFPE	3h	16207	22319	47.0	11.5	118	51.7	50.5	2.45	1.47	32.1
60-3	Colon	FFPE	12h	16172	21860	46.9	12.8	186	58.9	57.5	2.27	1.86	35.1
74-10	Kidney	Frozen	Ctl	17291	21732	50.1	8.8	318	62.2	60.5	2.65	0.99	65.4
74-1	Kidney	FFPE	1h	15439	22925	44.8	20.0	139	63.1	61.4	2.73	1.65	29.6
74-2	Kidney	FFPE	2h	14499	22540	42.0	24.8	126	64.2	62.7	2.28	1.17	24.8
74-3	Kidney	FFPE	3h	15397	22763	44.6	20.0	138	63.8	62.3	2.34	1.90	30.1
74-4	Kidney	FFPE	12h	15290	22780	44.3	18.3	117	53.2	51.8	2.58	1.59	27.4
88-10	Kidney	Frozen	Ctl	17689	22131	51.3	8.7	298	60.3	58.8	2.4	0.74	62.3
88-1	Kidney	FFPE	1h	15261	22916	44.2	23.4	138	62.2	60.6	2.65	1.78	28.2
88-2	Kidney	FFPE	2h	15479	22617	44.9	20.0	135	60.0	58.6	2.39	2.07	28.7
88-3	Kidney	FFPE	3h	15594	22717	45.2	21.1	144	63.5	62.0	2.35	2.10	28.9
88-4	Kidney	FFPE	12h	15988	24722	46.4	19.1	111	60.9	59.4	2.43	2.89	32.1

Supplemental Table 3: Results of pathway analysis of genes showing fixation-related changes in expression using Database for Annotation, Visualization and Integrated Discovery (DAVID).

Category	Term	Count	%	p-value	Benjamini-Hochberg correction
GOTERM_CC_DIRECT	Nucleosome	39	2.76	2.54E-22	1.37E-19
UP_KEYWORDS	Nucleosome core	37	2.62	4.89E-21	2.27E-18
INTERPRO	Histone-fold	40	2.83	3.72E-20	5.85E-17
UP_KEYWORDS	Citrullination	36	2.55	1.34E-18	3.11E-16
KEGG_PATHWAY	Systemic lupus erythematosus	41	2.90	6.30E-18	1.56E-15
INTERPRO	Histone core	28	1.98	4.78E-16	3.49E-13
KEGG_PATHWAY	Alcoholism	43	3.04	9.41E-15	1.17E-12
GOTERM_BP_DIRECT	Nucleosome assembly	33	2.33	3.81E-13	1.10E-09
GOTERM_BP_DIRECT	Telomere organization	15	1.06	7.51E-11	1.09E-07
GOTERM_BP_DIRECT	Chromatin silencing at rDNA	17	1.20	1.08E-10	1.04E-07
GOTERM_BP_DIRECT	DNA replication-dependent nucleosome assembly	15	1.06	1.33E-09	9.60E-07
UP_SEQ_FEATURE	Cross-link:Glycyl lysine isopeptide (Lys-Gly) (interchain with G-Cter in ubiquitin)	42	2.97	8.84E-09	2.96E-05
GOTERM_BP_DIRECT	Protein heterotetramerization	16	1.13	9.86E-09	5.71E-06
GOTERM_MF_DIRECT	Protein heterodimerization activity	60	4.24	1.48E-08	1.37E-05
GOTERM_BP_DIRECT	Negative regulation of gene expression, epigenetic	17	1.20	1.95E-08	9.41E-06
GOTERM_CC_DIRECT	Nuclear nucleosome	16	1.13	2.05E-08	3.69E-06
GOTERM_CC_DIRECT	Nuclear chromosome	17	1.20	4.96E-08	6.69E-06
INTERPRO	Histone H4, conserved site	9	0.64	3.67E-07	1.92E-04

Supplementary Table 4: Basic miRNA summary data organized by sample. The complete dataset is available through dbGaP (phs001639).

Specimen ID	Tissue	Pres Type	DTF	# miRNA Detected (>=3 counts)	Median count	max count miRNA	total clusters (Mil)	Mean Trimmed Length	% Mapped to Genome	%(G+C) aligned	(C/T) ratio
1-10	Kidney	Frozen	Ctl	387	48	hsa-miR-26a-5p	6.09	24.2	94.92	46.74	0.79
1-2	Kidney	FFPE	1h	345	40	hsa-miR-26a-5p	5.9	27.7	90.51	49.34	0.89
1-4	Kidney	FFPE	2h	364	46	hsa-miR-143-3p	6.1	26.9	91.21	49.15	0.87
1-1	Kidney	FFPE	3h	375	39	hsa-miR-486-5p	5.69	25.8	91.35	49.28	0.87
1-3	Kidney	FFPE	12h	341	39	hsa-miR-26a-5p	6.22	27.5	89.68	49.62	0.86
2-10	Kidney	Frozen	Ctl	402	88	hsa-miR-148a-3p	6.48	24.3	94.37	46.87	0.82
2-1	Kidney	FFPE	1h	382	79.5	hsa-miR-148a-3p	6.24	25.5	91.87	49.17	0.9
2-3	Kidney	FFPE	2h	373	69	hsa-miR-148a-3p	6.39	27.3	90.46	49.6	0.91
2-2	Kidney	FFPE	3h	379	82	hsa-miR-148a-3p	6.68	25.7	90.66	50.34	0.94
2-4	Kidney	FFPE	12h	363	71	hsa-miR-148a-3p	6.61	26.2	89.59	50.03	0.92
3-1	Kidney	Frozen	Ctl	344	36	hsa-miR-26a-5p	5.81	27.7	85.37	50.22	0.9
3-4	Kidney	FFPE	1h	378	43	hsa-miR-21-5p	6.16	27.4	90.95	48.82	0.87
3-3	Kidney	FFPE	2h	298	42	hsa-miR-21-5p	5.79	30.4	86.56	50.32	0.89
3-2	Kidney	FFPE	3h	328	37	hsa-miR-21-5p	6.48	28.9	87.15	49.48	0.87
3-10	Kidney	FFPE	12h	420	53.5	hsa-miR-21-5p	6.8	24.4	95.14	45.28	0.74
4-10	Kidney	Frozen	Ctl	393	48	hsa-miR-26a-5p	6.17	23.8	94.53	46.57	0.79
4-1	Kidney	FFPE	1h	362	50.5	hsa-miR-26a-5p	6.2	24.9	93.23	46.83	0.81
4-2	Kidney	FFPE	2h	328	49.5	hsa-miR-26a-5p	6.37	27.4	90.76	48.41	0.85
4-3	Kidney	FFPE	3h	336	46	hsa-miR-143-3p	6.24	27.4	88.49	48.57	0.84
4-4	Kidney	FFPE	12h	258	38	hsa-miR-143-3p	5.98	30.3	85.55	50.95	0.89
5-10	Kidney	Frozen	Ctl	365	67	hsa-miR-26a-5p	6.1	24.8	94.22	45.72	0.76
5-1	Kidney	FFPE	1h	386	47	hsa-miR-143-3p	5.69	25	92.98	46.76	0.8
5-4	Kidney	FFPE	2h	370	48.5	hsa-miR-26a-5p	6.92	26.1	93.02	47.15	0.81
5-3	Kidney	FFPE	3h	349	57	hsa-miR-26a-5p	6.38	27	90.77	48.09	0.83
5-2	Kidney	FFPE	12h	343	54	hsa-miR-486-5p	6.41	26.5	89.94	48.97	0.86
7-10	Kidney	Frozen	Ctl	407	52	hsa-miR-26a-5p	6.99	23.5	96.7	45.91	0.77
7-2	Kidney	FFPE	1h	369	52	hsa-miR-26a-5p	6.28	23.1	96.03	45.38	0.78
7-3	Kidney	FFPE	2h	370	48	hsa-miR-26a-5p	7.09	24.5	93.38	46.3	0.79
7-4	Kidney	FFPE	3h	364	54	hsa-miR-26a-5p	5.85	23	96.58	45.26	0.76
7-1	Kidney	FFPE	12h	383	48	hsa-miR-26a-5p	6.47	24.5	92.9	47.94	0.83
9-10	Kidney	Frozen	Ctl	387	42	hsa-miR-26a-5p	6.49	24.8	93.64	47.66	0.84
9-4	Kidney	FFPE	1h	376	48.5	hsa-miR-10b-5p	7.18	26.3	91.91	47.34	0.82
9-1	Kidney	FFPE	2h	374	53	hsa-miR-26a-5p	6.7	24.2	94.6	46.79	0.82
9-2	Kidney	FFPE	3h	384	50.5	hsa-miR-26a-5p	6.63	24.4	94.2	46.44	0.8

Specimen ID	Tissue	Pres Type	DTF	# miRNA Detected (>=3 counts)	Median count	max count miRNA	total clusters (Mil)	Mean Trimmed Length	% Mapped to Genome	%(G+C) aligned	(C/T) ratio
9-3	Kidney	FFPE	12h	338	53.5	hsa-miR-26a-5p	6.82	26.6	89.88	48.22	0.83
10-10	Kidney	Frozen	Ctl	412	50.5	hsa-miR-26a-5p	6.74	24.9	94.14	46.53	0.79
10-1	Kidney	FFPE	1h	399	50	hsa-miR-26a-5p	6.32	24.2	94.1	46.63	0.8
10-4	Kidney	FFPE	2h	408	46.5	hsa-miR-26a-5p	7.62	25.2	93.46	47.3	0.81
10-3	Kidney	FFPE	3h	333	45	hsa-miR-26a-5p	6.26	27.3	90.85	48.08	0.81
10-2	Kidney	FFPE	12h	386	47	hsa-miR-26a-5p	6.33	26.3	92.39	47.77	0.82
41-10	Ovary	Frozen	Ctl	417	48	hsa-miR-182-5p	6.86	24.6	94.49	45.01	0.78
41-3	Ovary	FFPE	1h	388	56	hsa-miR-26a-5p	6.89	25.6	93.26	45.47	0.78
41-2	Ovary	FFPE	2h	379	45	hsa-miR-182-5p	6.78	27.7	91.53	47.35	0.84
41-1	Ovary	FFPE	3h	392	45	hsa-miR-26a-5p	7.12	26.7	91.99	47.13	0.83
41-4	Ovary	FFPE	12h	373	46	hsa-miR-182-5p	6.37	26.7	91.58	47.21	0.84
42-10	Ovary	Frozen	Ctl	485	70	hsa-miR-26a-5p	7.16	24.7	94.89	45.27	0.75
42-4	Ovary	FFPE	1h	469	57	hsa-miR-26a-5p	6.49	24.8	94.58	46.23	0.8
42-1	Ovary	FFPE	2h	480	54.5	hsa-miR-92a-3p	6.38	24.1	94.5	46.61	0.83
42-2	Ovary	FFPE	3h	506	64	hsa-miR-26a-5p	6.86	24.6	94.27	47.07	0.83
42-3	Ovary	FFPE	12h	499	56	hsa-miR-26a-5p	6.48	24.3	94.51	46.27	0.81
43-10	Colon	Frozen	Ctl	441	38	hsa-miR-143-3p	6.83	24.3	88.61	50.42	0.94
43-3	Colon	FFPE	1h	449	42	hsa-miR-21-5p	6.01	24.9	91.56	49.11	0.89
43-1	Colon	FFPE	2h	430	44	hsa-miR-21-5p	6.34	25.2	91.73	49.53	0.91
43-2	Colon	FFPE	3h	413	41	hsa-miR-21-5p	5.51	26.1	86.46	53.05	1.08
43-4	Colon	FFPE	12h	355	33	hsa-miR-143-3p	6.03	24.2	77.96	61.77	1.54
44-10	Colon	Frozen	Ctl	417	52	hsa-miR-143-3p	6.92	25.4	93.05	47.23	0.79
44-2	Colon	FFPE	1h	424	49	hsa-miR-192-5p	6.33	26.6	89.69	48.43	0.87
44-4	Colon	FFPE	2h	382	43	hsa-miR-192-5p	6.01	27.8	93.21	48.05	0.83
44-1	Colon	FFPE	3h	404	46	hsa-miR-192-5p	6.24	26	88.25	50.11	0.93
44-3	Colon	FFPE	12h	317	45	hsa-miR-192-5p	5.99	28.2	81.9	52.25	1.02
45-10	Colon	Frozen	Ctl	432	41	hsa-miR-192-5p	6.2	25	92.47	48.54	0.87
45-1	Colon	FFPE	1h	427	46	hsa-miR-192-5p	6.12	23.5	94.78	47.01	0.81
45-2	Colon	FFPE	2h	436	48	hsa-miR-192-5p	6.84	26.3	91.06	48.69	0.9
45-3	Colon	FFPE	3h	417	37	hsa-miR-192-5p	6.56	25	92.15	49.59	0.89
45-4	Colon	FFPE	12h	401	37	hsa-miR-192-5p	6.44	24.6	88.48	51.15	0.98
46-10	Ovary	Frozen	Ctl	450	61.5	hsa-miR-26a-5p	7.07	23.4	96.21	45.02	0.74
46-1	Ovary	FFPE	1h	412	46	hsa-miR-26a-5p	6.86	24.6	94.6	46.33	0.8
46-4	Ovary	FFPE	2h	426	54	hsa-miR-26a-5p	6.67	24.8	93.98	47.2	0.82
46-2	Ovary	FFPE	3h	384	36	hsa-miR-26a-5p	6.26	27.4	92.49	48.24	0.85
46-3	Ovary	FFPE	12h	434	47	hsa-miR-26a-5p	6.1	24.1	93.37	47.46	0.82
47-10	Ovary	Frozen	Ctl	459	49	hsa-miR-26a-5p	7.42	25.5	91.38	47.59	0.83
47-1	Ovary	FFPE	1h	485	54	hsa-miR-26a-5p	6.93	23.8	94.32	47.09	0.81

Specimen ID	Tissue	Pres Type	DTF	# miRNA Detected (>=3 counts)	Median count	max count miRNA	total clusters (Mil)	Mean Trimmed Length	% Mapped to Genome	%(G+C) aligned	(C/T) ratio
47-4	Ovary	FFPE	2h	453	51	hsa-miR-26a-5p	7.15	23.9	93.3	48.06	0.86
47-3	Ovary	FFPE	3h	485	55	hsa-miR-26a-5p	7.34	23.9	93.5	47.32	0.83
47-2	Ovary	FFPE	12h	388	38	hsa-miR-26a-5p	6.68	27.7	84.31	51.23	0.96
48-10	Ovary	Frozen	Ctl	416	53.5	hsa-miR-26a-5p	6.84	23.9	94.49	45.05	0.78
48-3	Ovary	FFPE	1h	398	39.5	hsa-miR-92a-3p	7.02	24.3	93.32	47.36	0.85
48-4	Ovary	FFPE	2h	418	62.5	hsa-miR-10a-5p	7.84	22.9	95.9	45.3	0.81
48-1	Ovary	FFPE	3h	421	52	hsa-miR-92a-3p	7.13	24.2	92.99	46.56	0.83
48-2	Ovary	FFPE	12h	397	51	hsa-miR-26a-5p	7.37	25.8	89.9	47.23	0.82
49-10	Ovary	Frozen	Ctl	462	48.5	hsa-miR-26a-5p	7.14	24.6	94.71	45.48	0.74
49-3	Ovary	FFPE	1h	428	42.5	hsa-miR-92a-3p	6.1	26.5	90.45	50.1	0.92
49-4	Ovary	FFPE	2h	422	51.5	hsa-miR-26a-5p	6.73	26.4	91.95	48.46	0.85
49-2	Ovary	FFPE	3h	347	49	hsa-miR-26a-5p	6.51	29.5	88.05	49.2	0.85
49-1	Ovary	FFPE	12h	323	35	hsa-miR-92a-3p	6.64	30.4	88.09	50.31	0.92
50-10	Ovary	Frozen	Ctl	407	46	hsa-miR-92a-3p	7.2	24.8	92.67	49.77	0.89
50-4	Ovary	FFPE	1h	434	55.5	hsa-miR-26a-5p	7.37	24.2	93.43	47.88	0.83
50-2	Ovary	FFPE	2h	440	47	hsa-miR-92a-3p	7.51	24.5	92.39	49.2	0.88
50-3	Ovary	FFPE	3h	414	50.5	hsa-miR-92a-3p	6.56	23.3	94.68	47.51	0.82
50-1	Ovary	FFPE	12h	427	52	hsa-miR-92a-3p	6.27	23.4	91.85	51.13	0.96
51-10	Colon	Frozen	Ctl	434	51	hsa-miR-143-3p	6.94	23.5	95.54	46.3	0.77
51-4	Colon	FFPE	1h	375	34	hsa-miR-143-3p	5.04	23.2	89.39	51.6	0.99
51-2	Colon	FFPE	2h	414	49	hsa-miR-143-3p	5.88	25.1	91.61	49.07	0.87
51-1	Colon	FFPE	3h	412	41	hsa-miR-143-3p	5.95	26	90.83	49.86	0.89
51-3	Colon	FFPE	12h	375	39	hsa-miR-21-5p	7.5	24.8	82.81	55.76	1.18
52-10	Colon	Frozen	Ctl	396	59.5	hsa-miR-26a-5p	5.83	23.3	94.86	46.54	0.79
52-1	Colon	FFPE	1h	403	43	hsa-miR-192-5p	5.01	22.9	93.58	49.24	0.91
52-2	Colon	FFPE	2h	429	47	hsa-miR-21-5p	5.57	22.9	94.73	47.65	0.84
52-3	Colon	FFPE	3h	415	47	hsa-miR-143-3p	5.51	23.3	95.02	46.76	0.79
52-4	Colon	FFPE	12h	384	45	hsa-miR-21-5p	5.85	25.7	89.74	50.17	0.91
53-10	Ovary	Frozen	Ctl	451	51	hsa-miR-26a-5p	6.14	25.7	93.02	46.74	0.8
53-2	Ovary	FFPE	1h	473	54	hsa-miR-21-5p	6.36	23.5	94.89	47.58	0.84
53-3	Ovary	FFPE	2h	397	51	hsa-miR-21-5p	5.8	27.3	92.8	47.67	0.85
53-4	Ovary	FFPE	3h	394	46	hsa-miR-21-5p	5.8	28.7	90.84	48.26	0.86
53-1	Ovary	FFPE	12h	405	42	hsa-miR-21-5p	6.27	27.9	90.09	48.43	0.85
54-10	Colon	Frozen	Ctl	421	60	hsa-miR-192-5p	6.68	25.3	94.57	46.91	0.78
54-2	Colon	FFPE	1h	403	44	hsa-miR-192-5p	5.43	26	92.9	48.34	0.84
54-3	Colon	FFPE	2h	379	50	hsa-miR-192-5p	5.98	27.3	91.02	48.4	0.84
54-1	Colon	FFPE	3h	381	34	hsa-miR-192-5p	5.49	27.3	90.25	49.68	0.88
54-4	Colon	FFPE	12h	272	36	hsa-miR-192-5p	5.79	28.7	76.56	54.73	1.14

Specimen ID	Tissue	Pres Type	DTF	# miRNA Detected (>=3 counts)	Median count	max count miRNA	total clusters (Mil)	Mean Trimmed Length	% Mapped to Genome	%(G+C) aligned	(C/T) ratio
55-10	Ovary	Frozen	Ctl	371	46	hsa-miR-10b-5p	6.36	24.3	92.82	45.73	0.79
55-1	Ovary	FFPE	1h	373	38	hsa-miR-10b-5p	6.38	24.4	93	46.43	0.81
55-2	Ovary	FFPE	2h	353	41	hsa-miR-10b-5p	5.92	25.4	90.78	48.03	0.87
55-3	Ovary	FFPE	3h	351	42	hsa-miR-10b-5p	5.61	24.4	94.06	45.21	0.77
55-4	Ovary	FFPE	12h	341	37	hsa-miR-10b-5p	5.48	24	93.7	46.21	0.8
56-10	Colon	Frozen	Ctl	434	49	hsa-miR-192-5p	6.25	23.9	92.77	46.72	0.8
56-3	Colon	FFPE	1h	347	40	hsa-miR-192-5p	6.45	24.8	77.77	56.33	1.24
56-4	Colon	FFPE	2h	353	43	hsa-miR-192-5p	5.49	28	89.06	48.54	0.85
56-1	Colon	FFPE	3h	413	44	hsa-miR-192-5p	5.48	24.3	92.11	47.85	0.84
56-2	Colon	FFPE	12h	409	45	hsa-miR-192-5p	6.22	24.6	90.52	48.66	0.87
57-10	Colon	Frozen	Ctl	386	47.5	hsa-miR-192-5p	5.67	26.1	90.45	46.97	0.78
57-1	Colon	FFPE	1h	398	50	hsa-miR-192-5p	6.75	25.3	90.59	47.74	0.82
57-4	Colon	FFPE	2h	377	50	hsa-miR-192-5p	5.67	24.3	90.41	48.41	0.84
57-3	Colon	FFPE	3h	343	39	hsa-miR-192-5p	5.04	26.4	87.86	49.12	0.87
57-2	Colon	FFPE	12h	254	33	hsa-miR-192-5p	5.88	27.2	73.57	56.32	1.23
58-10	Colon	Frozen	Ctl	407	58	hsa-miR-192-5p	6.51	23	95.93	44.91	0.71
58-1	Colon	FFPE	1h	402	47.5	hsa-miR-192-5p	5.56	23.1	92.55	47.65	0.84
58-2	Colon	FFPE	2h	393	41	hsa-miR-192-5p	5.43	23.6	91.98	48.19	0.85
58-3	Colon	FFPE	3h	372	54	hsa-miR-192-5p	5.88	24.6	91.95	47.59	0.82
58-4	Colon	FFPE	12h	387	45	hsa-miR-192-5p	5.22	24	91.56	47.99	0.84
59-10	Ovary	Frozen	Ctl	400	55	hsa-miR-26a-5p	6.56	23.7	95.51	44.49	0.74
59-1	Ovary	FFPE	1h	426	48	hsa-miR-21-5p	7.44	25.2	91.86	46.46	0.81
59-2	Ovary	FFPE	2h	388	46	hsa-miR-143-3p	6.17	25.9	89.42	48.2	0.86
59-3	Ovary	FFPE	3h	385	47	hsa-miR-143-3p	5.56	24.9	90.38	48.31	0.87
59-4	Ovary	FFPE	12h	383	49	hsa-miR-143-3p	5.64	23.7	90.36	49.73	0.91
60-10	Colon	Frozen	Ctl	455	49	hsa-miR-192-5p	6.72	23.1	96.26	45.32	0.75
60-2	Colon	FFPE	1h	400	38	hsa-miR-192-5p	6.3	24.9	93.97	47.03	0.84
60-4	Colon	FFPE	2h	388	37	hsa-miR-192-5p	6.01	26.5	93.45	47.39	0.84
60-1	Colon	FFPE	3h	394	40.5	hsa-miR-192-5p	6.19	24.1	92.98	47.5	0.86
60-3	Colon	FFPE	12h	404	41	hsa-miR-192-5p	6.28	24.6	93.08	47.94	0.86
74-10	Kidney	Frozen	Ctl	402	44	hsa-miR-26a-5p	6.7	23.2	96.08	45.68	0.77
74-1	Kidney	FFPE	1h	370	45	hsa-miR-26a-5p	7.25	25	94.38	47.36	0.82
74-2	Kidney	FFPE	2h	383	48	hsa-miR-26a-5p	6.82	23.9	95.6	46.6	0.79
74-3	Kidney	FFPE	3h	354	44	hsa-miR-26a-5p	6.72	25.8	95.13	47.78	0.81
74-4	Kidney	FFPE	12h	393	47	hsa-miR-26a-5p	7.51	24.1	95.05	47.61	0.83
88-10	Kidney	Frozen	Ctl	418	46.5	hsa-miR-486-5p	7.66	25.5	94.37	47.9	0.83
88-1	Kidney	FFPE	1h	375	40	hsa-miR-486-5p	6.6	23.1	96.47	47.26	0.83
88-2	Kidney	FFPE	2h	411	52	hsa-miR-486-5p	7.37	24.1	93.93	47.41	0.81

Specimen ID	Tissue	Pres Type	DTF	# miRNA Detected (>=3 counts)	Median count	max count miRNA	total clusters (Mil)	Mean Trimmed Length	% Mapped to Genome	%(G+C) aligned	(C/T) ratio
88-3	Kidney	FFPE	3h	412	48.5	hsa-miR-486-5p	8.37	24.6	92.25	47.83	0.82
88-4	Kidney	FFPE	12h	376	44.5	hsa-miR-486-5p	7.31	26	87.43	48.83	0.84
